# Making use of multiple surveys: Estimating breeding probability using a multievent‐robust design capture–recapture model

**DOI:** 10.1002/ece3.4828

**Published:** 2019-02-05

**Authors:** W. Chris Oosthuizen, Roger Pradel, Marthán N. Bester, P. J. Nico de Bruyn

**Affiliations:** ^1^ Department of Zoology and Entomology Mammal Research Institute University of Pretoria Hatfield South Africa; ^2^ Biostatistics and Population Biology Group CEFE, CNRS, Univ Montpellier, Univ Paul Valéry Montpellier 3, EPHE, IRD Montpellier France

**Keywords:** auxiliary data, breeding propensity, elephant seal, incidental observations, intermittent breeding, robust design, unobservable state

## Abstract

Increased environmental stochasticity due to climate change will intensify temporal variance in the life‐history traits, and especially breeding probabilities, of long‐lived iteroparous species. These changes may decrease individual fitness and population viability and is therefore important to monitor. In wild animal populations with imperfect individual detection, breeding probabilities are best estimated using capture–recapture methods. However, in many vertebrate species (e.g., amphibians, turtles, seabirds), nonbreeders are unobservable because they are not tied to a territory or breeding location. Although unobservable states can be used to model temporary emigration of nonbreeders, there are disadvantages to having unobservable states in capture–recapture models. The best solution to deal with unobservable life‐history states is therefore to eliminate them altogether. Here, we achieve this objective by fitting novel multievent‐robust design models which utilize information obtained from multiple surveys conducted throughout the year. We use this approach to estimate annual breeding probabilities of capital breeding female elephant seals (*Mirounga leonina*). Conceptually, our approach parallels a multistate version of the Barker/robust design in that it combines robust design capture data collected during discrete breeding seasons with observations made at other times of the year. A substantial advantage of our approach is that the nonbreeder state became “observable” when multiple data sources were analyzed together. This allowed us to test for the existence of state‐dependent survival (with some support found for lower survival in breeders compared to nonbreeders), and to estimate annual breeding transitions to and from the nonbreeder state with greater precision (where current breeders tended to have higher future breeding probabilities than nonbreeders). We used program E‐SURGE (2.1.2) to fit the multievent‐robust design models, with uncertainty in breeding state assignment (breeder, nonbreeder) being incorporated via a hidden Markov process. This flexible modeling approach can easily be adapted to suit sampling designs from numerous species which may be encountered during and outside of discrete breeding seasons.

## 1 INTRODUCTION

Intermittent breeding is widespread among vertebrate taxa (e.g., fish: Jørgensen, Ernande, Fiksen, & Dieckmann, 2006, amphibians: Muths, Scherer, & Lambert, 2010, reptiles: Baron, Le Galliard, Ferrière, & Tully, 2013, birds: Cam, Hines, Monnat, Nichols, & Danchin, 1998 and mammals: Pilastro, Tavecchia, & Marin, 2003). Some long‐lived iteroparous species such as biennially breeding albatross (Barbraud & Weimerskirch, 2012) mostly follow strictly intermittent breeding tactics due to the long time required to raise offspring and the need to sequester body condition necessary for successful breeding at a subsequent occasion. Many other long‐lived species are prone to annual reproduction, but females may intermittently fail to breed because of impairment during the early stages of the reproductive cycle. The proximate regulator of intermittent breeding is thought to closely couple with an individual's energy balance, with reproductive skipping more prevalent among individuals in poor condition (Drent & Daan, 1980). At an ultimate level, females face a fitness trade‐off between current and future reproduction (Reed, Harris, & Wanless, 2015). Adaptive explanations of intermittent breeding postulate that, under certain circumstances, reproductive skipping may increase average lifetime reproductive success above what could be achieved through persistent attempts at annual reproduction (Bull & Shine, 1979). According to the prudent parent hypothesis (Drent & Daan, 1980), intermittent breeding is adaptive when the energetic savings associated with skipped breeding opportunities can be diverted to improve an individual's survival probability or subsequent fecundity. In contrast, nonadaptive explanations suggest that intermittent breeding is itself not advantageous, but instead an unavoidable consequence of ecological or social constraints.

Reliable estimates of breeding probability (the probability that a sexually mature individual will attempt to breed in a given year) are fundamental to the study of population dynamics in species exhibiting intermittent breeding. At the population level, breeding probability directly affects the number of births each year and so population growth rate (Nichols, Hines, Pollock, Hinz, & Link, 1994). Temporal fluctuations in breeding probability may drive population change even in long‐lived species at the late‐maturing and slow‐reproducing end of the slow‐fast continuum of life histories, despite its weak elasticity (Jenouvrier, Barbraud, Cazelles, & Weimerskirch, 2005). At the individual level, determining whether the covariance between breeding probability and survival is shaped by reproductive costs, individual heterogeneity, and/or environmental variation is of central importance to life‐history theory. But, breeding probability is one of the most difficult reproductive parameters to estimate reliably and is perhaps the least understood demographic process influencing annual fecundity (Etterson et al., 2011). As such, there still exists a need to refine our understanding of the individual fitness consequences of intermittent breeding, and whether intermittent breeding is an adaptive resource allocation (or life history) strategy under direct selection pressure, rather than a nonadaptive consequence of constraint (Reed et al., 2015).

The empirical estimation of breeding probability integrates information from both the breeding‐ and nonbreeding component of the population at every time step. In wild animal populations, imperfect observational sampling frequently presents a double dilemma to this estimation. Firstly, nonbreeders often avoid breeding sites in both terrestrial (amphibians: Church, Bailey, Wilbur, Kendall, & Hines, 2007; Muths et al., 2010) and marine species (turtles: Rivalan et al., 2005; seabirds: Converse, Kendall, Doherty, & Ryan, 2009; pinnipeds: De Bruyn et al., 2011), rendering them unobservable. Secondly, in most studies of natural populations in the wild some breeders escape detection even when present in the sampling area. As a consequence, failure to observe an individual may be because either the individual was a nonbreeder, or the individual was present and breeding, but escaped detection. An appropriate statistical framework accounting for imperfect detection (multistate capture–recapture methods and generalizations of such models; Lebreton, Nichols, Barker, Pradel, & Spendelow, 2009; Nichols et al., 1994; Pradel, 2009) is thus required to infer breeding probability in wild animal populations.

In this study, we use a novel multievent‐robust design approach to estimate breeding probability in a population of southern elephant seals (*Mirounga leonina*) (hereafter elephant seals). Like in many other synchronously reproducing vertebrates, nonbreeding elephant seals are mostly absent from breeding colonies (corresponding to temporary emigration during the breeding season). Nonbreeding elephant seals may, however, be observed outside of the breeding season, although these “auxiliary resightings” are uninformative with regard to the most recent reproductive state of an individual. Our intention is to show a flexible analytic approach where information obtained from multiple surveys conducted within and outside of the breeding season is combined to improve parameter estimation of breeding probability in particular. Conceptually, the model we use parallels a multistate version of the Barker/robust design (Kendall et al., 2013) in that it combines robust design capture data collected during discrete breeding seasons with observations made at other times of the year. Our aims were to (a) quantify the probability that an adult female elephant seal is a breeder in a given year, conditional on having bred once before, and surviving as part of the marked population; and (b) test whether breeding probability best corresponds to a random or Markovian process.

## 2 METHODS

### 2.1 Life cycle of female elephant seals

**Figure 1:**
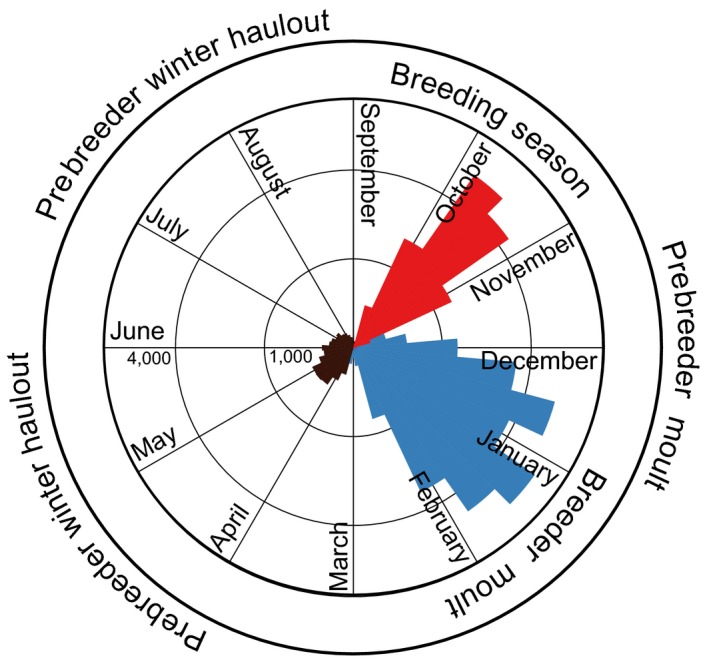
Annual cycle of female southern elephant seals symbolized by the temporal distribution of resights of individually marked southern elephant seals at Marion Island. Resights of weaned pups are excluded; values correspond to the cumulative number of resights per day (1984–2014). Adults are generally only observed during the breeding season (red bars) and the moult (blue bars). Prebreeders are observed in the moult and to a lesser extent, during the winter (brown bars)

Southern elephant seals are wide‐ranging marine predators with a circumpolar Southern Ocean distribution (Le Boeuf & Laws, 1994). They are the largest living pinnipeds and exhibit significant sexual dimorphism. The mating system is extreme polygyny with capital breeding females aggregating ashore in harems during a synchronous annual breeding season in the austral spring (Figure 1). Breeding females arrive and depart in a staggered fashion and numbers ashore approximate a normal distribution (Hindell & Burton, 1988, Supporting Information Figure S1 in Appendix S1). Pregnant females begin to arrive ashore in mid‐September, giving birth soon after arrival at the colony. Females remain ashore for the entire ~23‐day lactation period, nursing their pups daily. Four weeks after arrival at the colony, a female will mate with the dominant male and abruptly wean her pup before returning to sea (Le Boeuf & Laws, 1994). Female numbers peak around 15 October, and all breeding females have returned to sea by mid‐November. Nonbreeding females (prebreeders and adult females skipping reproduction) typically do not attend breeding aggregations and probably mate at sea (De Bruyn et al., 2011). Nonbreeders are thus absent from the breeding colony and unobservable at this time of the year—a form of temporary emigration.

All elephant seals older than young of the year are obliged to moult ashore for a month or more every summer. Prebreeders return to moult at the conclusion of the breeding season, whereas breeding females moult approximately 2 months after weaning their pups (Kirkman et al., 2003, Figure 1, Supporting Information Figures S2 and S3 in Appendix S1). Every individual moults for 4–5 weeks, during which time they mostly stay ashore. After the moult, adult seals return to and typically remain at sea to forage up until the next breeding season. In contrast, prebreeders frequently haul out and remain ashore for 2–4 weeks during the austral winter (Kirkman et al., 2001, Figure 1, Supporting Information Figure S2 in Appendix S1).

### 2.2 Field methods

From 1983 to 2009, virtually all (*n* = 6,439) recently weaned female elephant seal pups born at Marion Island in the southern Indian Ocean were marked with two unique livestock tags attached to the interdigital webbing of each hind flipper (Pistorius, de Bruyn, & Bester, 2011). In all breeding seasons from 1986 to 2013, trained observers resighted tagged seals at weekly intervals at all sites where elephant seals may breed. Given that breeding females remain ashore for approximately 4 weeks, opportunity exists to recapture a breeding female on multiple occasions within a breeding season. Additional to weekly breeding season surveys, observers conducted tag‐resights at all beaches every 10 days during the nonbreeding period (1984–2014, from November when the moult starts through to August, at the end of winter). Females of all reproductive states (prebreeders, breeders and nonbreeding adults) are observed during the moult (Kirkman et al., 2003), but reproductive states cannot be assigned to females seen moulting. Breeding season resights (*n* = 15,608) constituted only 27% of the total resights of female elephant seals made during the study period. Tagged adult females were encountered 13,911 times in the moult and prebreeder (juvenile) encounters during the winter and moult amounted to 28,224 resights.

### 2.3 Multievent‐robust design: general framework

In multistate capture–recapture models, individuals transition among a finite number of states, or die, according to a finite Markov chain (Lebreton et al., 2009). The capture probability of survivors is imperfect and can be modeled as the realization of a Bernoulli trial at each discrete time occasion. Given detection, the state occupied is assumed to be identified without error. In contrast, multievent models (Pradel, 2005) are hidden Markov models that describe two parallel processes: transitions among states, and the generation of observations (“events”) that relate to the true state that an individual occupies at each sampling occasion in a probabilistic framework (Gimenez, Lebreton, Gaillard, Choquet, & Pradel, 2012; Pradel, 2009). Thus, by separating the dynamics of the true state from the observation process, state uncertainty can be accommodated (Gimenez et al., 2012). In the elephant seal case, multievent models offer an efficient way to model survey data collected throughout the year, including observations with uncertain reproductive state. Integrating data collected during both the breeding and nonbreeding seasons negates the need for an unobservable nonbreeder state, which should increase the precision and accuracy of demographic parameter estimates (Kendall et al., 2009).

Robust design models (Kendall & Bjorkland, 2001; Kendall & Nichols, 1995; Pollock, 1982) differ from traditional capture–recapture models by distinguishing between primary and secondary sampling periods. Each primary period (e.g., a breeding season) comprises of a number of secondary occasions close together in time (e.g., multiple breeding season surveys). Because these secondary occasions provide a second source of information on capture probabilities, robust design often yields more identifiable parameters with less bias and better precision compared to traditional capture–recapture approaches (Kendall & Nichols, 2002). As with multievent models, state uncertainty can be incorporated in both the closed (Kendall, Hines, & Nichols, 2003) and open robust design (Cohen et al., 2018; Ruiz‐Gutierrez, Kendall, Saracco, & White, 2016) parameterizations. One of the generalizations of the classic robust design, the Barker/robust design (Kendall et al., 2013), combines recapture data collected under a robust design framework with auxiliary resightings or dead recoveries made at any time and place. The Barker/robust design is therefore an extension of Lindberg, Kendall, Hines, and Anderson (2001)'s robust design that incorporates not only data from dead recoveries, but also auxiliary live observations of marked individuals (Barker, 1997; Barker, Burnham, & White, 2004), to permit the joint modeling of survival, temporary and permanent emigration from a study area. However, although multistate and/or multievent extensions of the Barker/robust design are possible (Barker & White, 2004; Kendall et al., 2013), such models are not currently implemented in program MARK 9.0 (White & Burnham, 1999), the most widely used user‐friendly software tailored for capture–recapture analyses. The multievent‐robust design model we describe here is a general model that combines multistate open robust design capture data collected during discrete breeding seasons with auxiliary live observations made at other times of the year. We fitted the multievent‐robust design model using program E‐SURGE 2.1.2 (Choquet, Rouan, & Pradel, 2009), a user‐friendly software initially developed to analyze capture–recapture data as hidden Markov models.

### 2.4 Multievent‐robust design: the state process

The state process of a multievent‐robust design model is specified in the same way as traditional multistate or multievent models. Female elephant seals were assumed to occupy one of the following states each year: breeder (B, pupped in the current year); nonbreeder (NB, pupped previously, but not in the current year); and dead (D). The transition probabilities between states correspond to apparent annual survival (*ϕ*) (hereafter survival) and breeding probability (*ψ*). The state process can be decomposed as the product of a diagonal survival matrix by a conditional breeding probability matrix, with departure states in rows and arrival states in columns:

$$\left(\begin{array}{ccc} \varphi^{B} & 0 & 1-\varphi^{B}\\ 0 & \varphi^{\mathrm{NB}} & 1-\varphi^{\mathrm{NB}}\\ 0 & 0 & 1 \end{array}\right)\times \left(\begin{array}{ccc} \psi^{B‐B} & 1-\psi^{B‐B} & 0\\ \psi^{NB‐B} & 1-\psi^{NB‐B} & 0\\ 0 & 0 & 1 \end{array}\right).$$

For clarity of presentation we did not incorporate marker (tag) loss in the state process (Oosthuizen, Altwegg, Nevoux, Bester, & de Bruyn, 2018), although tag loss is known to occur at a low rate. As a consequence, the apparent survival estimates we obtain will be biased low. Rather than conditioning on survival only, the probability of breeding we estimate is conditional on the collective probability of survival and retention of at least one hind flipper tag.

### 2.5 Multievent‐robust design: the observation process

Capture–recapture field observations are typically summarized as an encounter history matrix. In its simplest form, an encounter history matrix is represented by a series of 0's (individual not observed) and 1's (individual observed). This matrix can be generalized to allow individuals to move across life‐history states over time (Lebreton & Pradel, 2002). In multistate models, the encounter history matrix is made up of a series of 0's and say, the integer values {1, 2}, designating for each capture occasion the state {1 = breeder, 2 = nonbreeder} in which the individual was encountered. To deal with uncertainty in the assignment of states, multistate models can be reformulated as multievent or state space models (Gimenez et al., 2012). Some of the field observations encoded in an encounter history matrix of a multievent model can be ambiguous, with the same observation code (event) corresponding to more than one state (though with potentially different probabilities).

**Figure 2:**
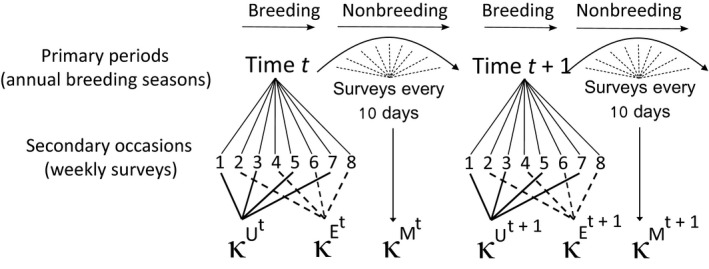
Robust design‐like structure of the model. We collapsed alternating secondary surveys within each breeding season (primary period) to generate two distinct capture periods (*κ* ^*U*^ and *κ* ^*E*^) per breeding season. Recaptures made outside of the breeding season were assigned to *κ* ^*M*^

In this study, encounter histories conditioned on the first encounter of a breeding female; the population of interest thus consisted of those seals that recruited to the breeding population and we did not estimate the probability of first reproduction. Each elephant seal breeding season comprised *j *=* *8 secondary surveys (the weekly island‐wide surveys). We collapsed alternating secondary surveys within each breeding season to generate two distinct capture periods (*κ*) per breeding season (Figure 2). Surveys conducted during “uneven” survey weeks (*j* = 1, 3, 5, 7) of the breeding season collapsed to generate capture period *U* (*κ* ^*U*^), whereas surveys conducted during “even” weeks (*j* = 2, 4, 6, 8) collapsed to capture period *E *(*κ* ^*E*^). Female elephant seals arrive and depart in a staggered fashion through a breeding season, and collapsing secondary surveys in this way ensured that every breeding female was exposed to survey effort in both *κ* ^*U*^ and *κ* ^*E*^. In contrast, if we had chosen to collapse the secondary occasions along the midpoint of the breeding season (e.g., *κ* ^1^ = *j*{1, 2, 3, 4} and *κ* ^2^ = *j*{5, 6, 7, 8}), females that entered the breeding season very early or after the peak may have been present and available for detection in only one of the capture periods, or at least would have been very heterogeneous in their availability for detection in each period. Thus, by collapsing alternating secondary surveys, we generated an approximately equal chance for all individuals to be captured in *κ* ^*U*^ and *κ* ^*E*^, given that all breeding females will be exposed to survey effort for approximately 2 weeks in each of *κ* ^*U*^ and *κ* ^*E*^.

**Figure 3:**
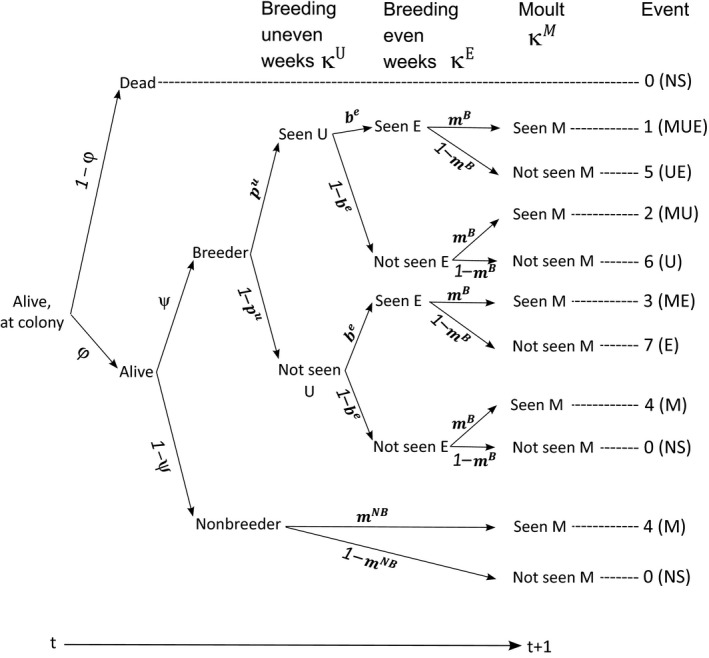
Fate diagram describing the probability structure of events for tagged elephant seals at Marion Island from the start of a breeding season in year *t* until the following breeding season in *t* + 1. The transition probabilities correspond to annual survival (*ϕ*) and annual breeding probability (*ψ*). The event probabilities correspond to the capture probability during “uneven” survey weeks of the breeding season (*p* ^*u*^), the capture probability during “even” survey weeks of the breeding season (*b* ^*e*^), and capture probabilities outside of the breeding season (*m*)

Within each breeding season, a breeding female could (a) be encountered during both capture periods *U* and *E* (*UE*); (b) only be encountered in *κ* ^*U*^ (*U*); (c) only be encountered in *κ* ^*E*^ (*E*); (d) not be encountered in either capture period (NS). All recaptures made outside of the breeding season (whether during the moult, winter or both these nonbreeding periods) were summarized as a single observation and assigned to capture period *M* (*κ* ^*M*^). A seal was therefore considered encountered during *κ* ^*M*^ if it was seen at least once during the winter or while moulting. We defined eight composite events (Table 1) by integrating resighting data collected for every individual within a “seal year” (September [*t*] to August [*t* + 1]) (i.e., during all three capture periods) as a single event. These eight composite events were encoded in the encounter history matrix of the multievent model. The encounter history matrix thus simply encodes the particular combination of field observations that was made, and not the states of the model (Figure 3).

**Table 1:** The eight possible events assigned to a female elephant seal and encoded in the encounter history matrix based on multiple capture periods in a year

Event code	Description	Index
0	Not seen during any capture period	*NS*
1	Seen in all three capture periods (*κ* ^*U*^, *κ* ^*E*^, *κ* ^*M*^)	*MUE*
2	Seen in *κ* ^*U*^ (breeding season “uneven” sampling weeks) and *κ* ^*M*^	*MU*
3	Seen in *κ* ^*E*^ (breeding season “even” sampling weeks) and *κ* ^*M*^	*ME*
4	Only seen in *κ* ^*M*^ (outside of the breeding season)	*M*
5	Seen in *κ* ^*U*^ and *κ* ^*E*^	*UE*
6	Only seen in *κ* ^*U*^	*U*
7	Only seen in *κ* ^*E*^	*E*

The observation process conditions on the underlying states, and was described via the product of three matrices. We defined the observation process occurring outside of the breeding season (*κ* ^*M*^) first. Our decision to model *κ* ^*M*^ first was purely practical, as the order of the three event matrices (***B*** ^***M***^, ***B*** ^***U***^ and ***B*** ^***E***^) has no influence on parameter estimates. The advantage of modeling *κ* ^*M*^ first comes to fruition in more general models that also include a prebreeder state, as this will reduce matrix dimensions compared to models specifying the breeding season matrices (***B*** ^***U***^ and ***B*** ^***E***^) first. When prebreeders are considered, specifying the breeding season matrices first would force us to have unique matrix columns for prebreeders and nonbreeders in each of ***B*** ^***U***^ and ***B*** ^***E***^ so that their capture probabilities outside the breeding season can vary. In contrast, when modeling capture probability outside of the breeding season first (matrix ***B*** ^***M***^), prebreeders and nonbreeders can enter the breeding season matrices as one category (i.e., in a single matrix column). The event matrix for *κ* ^*M*^ is

BM=MBMB¯MNBMNB¯B(mB1−mB00)NB00mNB1−mNBD0001

where ***B*** ^***M***^ is row‐stochastic (i.e., probabilities in a row sum to one) with states in rows and events in columns. Matrix ***B*** ^***M***^ gives the state‐specific probability of capture during the moult and winter. Horizontal lines (bars) above column index labels denote “not seen.” Column 1 (*M*_*B*_) represents breeders detected in *κ* ^*M*^; column 2 ($\bar{M_{B}}$) represents breeders not seen in *κ* ^*M*^; column 3 (*M*_*NB*_) represents nonbreeders detected in *κ* ^*M*^; and column 4 ($\bar{M_{\mathrm{NB}}}$) represents nonbreeders not seen in *κ* ^*M*^. A nonbreeder therefore has a probability ***m*** ^***NB***^ to be seen during either the moult or the winter, and the complement probability (**1** − ***m*** ^***NB***^) to escape detection.

Next, we defined the observation process during *κ* ^*U*^, that is, the “uneven” survey weeks of the breeding season.

BU=UMU¯MUU¯MNBMNB¯MB(pu1−pu0000)MB¯00pu1−pu00MNB000010MNB¯000001

The model assumes that nonbreeders are not available for encounter in the breeding season; rows 3 and 4 of matrix ***B*** ^***U***^ consequently have no free parameters. This assumption is plausible for elephant seals, but it can also be relaxed to include observable nonbreeders and state uncertainty within the breeding season. We present such an example in Supporting Information Appendix S2. Rows 1 and 2 of matrix ***B*** ^***U***^ give the probability ***p*** ^***u***^ that a breeder was captured during at least one “uneven” survey week; its compliment (**1 −** ***p*** ^***u***^) is the probability of not being encountered during any of the *j* weekly surveys of *κ* ^*U*^. Row 1 of the matrix corresponds to breeders also seen in *κ* ^*M*^; row 2 refers to breeders not detected in *κ* ^*M*^.

Lastly, we defined the observation process during the “even” survey weeks of the breeding season, where ***b*** ^***e***^ is the probability that a breeder was seen during any of the *j* weekly surveys of *κ* ^*E*^. The columns of matrix ***B*** ^***E***^ represent the events encountered in the field (Table 1).

BE=κEvent codeNS0MUE1MU2ME3M4UE5U6E7UM(0be1−be00000)U¯M000be1−be000U00000be1−be0U¯1−be000000beMNB00001000MNB¯10000000

### 2.6 Estimation of parameters and model selection

The state process of our umbrella model was $\varphi_{t}^{B\neq NB}\psi_{t}^{B-B\neq NB-B}$, that is, survival and breeding probabilities varying by time and reproductive state. We used quasi‐likelihood Akaike's Information Criterion (QAIC, Burnham & Anderson, 2002) and Akaike weights (*w*_*i*_) to compare this model to reduced‐parameter models in which first survival, and then, breeding probability was constrained with respect to time and/or reproductive state. QAIC was preferred as a model selection criterion to adjust parameter variances in light of marginal overdispersion (*ĉ* = 1.33) in the encounter histories (Supporting Information Appendix S3). The observation process part of the model was modeled as $p_{t}^{B\neq NB}$ in the nonbreeding period (*κ* ^*M*^) and $p_{t}^{B^{\kappa }}$during each of the breeding season capture periods *κ* ^*U*^ and *κ* ^*E*^. In other words, capture probabilities differed by time and reproductive state in the moult and winter, whereas breeding season capture probabilities varied by time and capture period.

## 3 RESULTS

**Figure 4:**
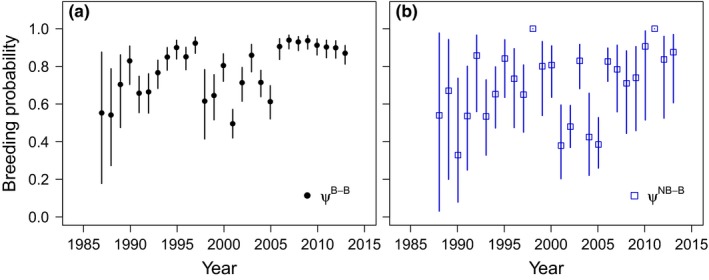
Annual breeding probability (mean and 95% confidence interval) of female elephant seals at Marion Island (1986 – 2013) estimated using a multievent‐robust design approach. (a) State transition from breeder to breeder ($\psi_{t}^{B-B}$) (b) State transition from nonbreeder to breeder ($\psi_{t}^{NB-B}$)

Two multievent‐robust design models received support in the data (Table 2). These models strongly indicated that breeding probabilities varied by time and previous breeding state (*w*_*i*_ = 0.99). Although the model with constant survival of adult females (φ = 0.76 [95% CI: 0.75–0.77]) was most parsimonious (*w*_*i*_ = 0.57), there was some support (*w*_*i*_ = 0.42) for lower survival in breeders (0.75 [0.74–0.76]) compared to nonbreeders (0.79 [0.75–0.82]). The probability of pupping in two consecutive years $\left(\psi_{t}^{B-B}\right)$ varied considerably between 1987 and 2005, but within a narrower range (mean 0.88–0.95) from 2006 to 2013 (Figure 4). Precision around $\psi_{t}^{B-B}$ was initially low, but improved as the number of cohorts and marked individuals in the breeding population increased. Transition probability from nonbreeder to breeder $\left(\psi_{t}^{NB-B}\right)$ had comparatively low precision in all years. The general tendency was that nonbreeders were less likely to give birth at *t* + 1 (*ψ* ^*NB*−*B*^ = 0.66 [0.61–0.71]) than females that were breeding in year *t* (*ψ* ^*BB*^ = 0.84 [0.82–0.86]).

**Table 2:** Comparison of multievent‐robust design models specifying different survival $\left(\varphi \right)$ and breeding $\left(\psi \right)$ probability patterns of southern elephant seals at Marion Island (1986–2013)

Model	np	Deviance	ΔQAIC	*w*_*i*_
$\varphi_{t}^{B\neq NB}\psi_{t}^{B-B\neq NB-B}$	214	23,754.06	56.70	0.00
$\varphi_{.}^{B\neq \mathrm{NB}}\psi_{t}^{B-B\neq \mathrm{NB}-B}$	**167**	**23,801.41**	**0.58**	**0.42**
$\varphi_{t}\psi_{t}^{B-B\neq NB-B}$	190	23,785.53	33.87	0.00
$\varphi_{.}\psi_{t}^{B-B\neq \mathrm{NB}-B}$	**166**	**23,803.19**	**0.00**	**0.57**
$\varphi_{.}\psi_{.}^{B-B\neq NB-B}$	115	24,151.12	176.34	0.00
$\varphi_{.}\psi_{t}$	140	23,878.31	8.09	0.01
$\varphi_{.}\psi_{.}$	114	24,205.87	218.14	0.00

The number of parameters (np), model deviance, ΔQAIC (the difference in QAIC between the model with the lowest QAIC value and the relevant model), and the relative support by the data of a model, in relation to the other models (QAIC weight, *w*_*i*_), is given. Models with support in the data are in boldface. Superscripts indicate state variation (*B *= breeder, *NB* = nonbreeder), subscripts identify the absence (.) or presence of time variation (*t*).

**Figure 5:**
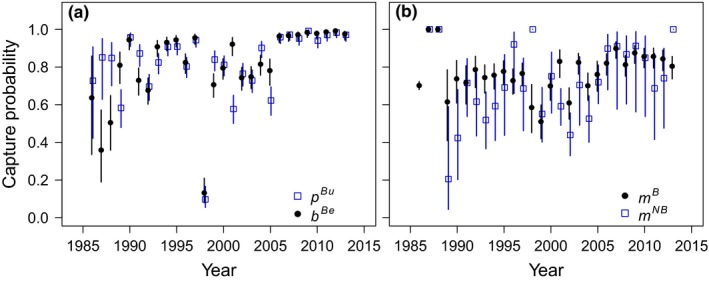
Capture probabilities (mean and 95% confidence interval) of female elephant seals at Marion Island estimated using a multievent‐robust design approach. (a) Capture probabilities (*p* ^*Bu*^; *b* ^*Be*^) of breeders in the “uneven” (*κ* ^*U*^) and “even” (*κ* ^*E*^) capture periods of every breeding season. (b) Capture probabilities of breeders (*m* ^*B*^, black circles) and nonbreeders (*m* ^*NB*^, blue squares) outside of the breeding season (i.e., through the moult and winter)

Capture probability was highest during the breeding season (poor detection in 1998 being a clear anomaly) and did not differ markedly between the two breeding season capture periods *κ* ^*U*^ and *κ* ^*E*^ (Figure 5). Both breeders and nonbreeders were encountered outside of the breeding season (nearly always in the moult, rather than the winter) with annual capture probabilities in capture period *κ* ^*M*^ often exceeding 0.7 (Figure 5).

## 4 DISCUSSION

Intermittent breeding is a widespread and important life‐history tactic among iteroparous species. In these species, breeding decisions have pervasive direct and indirect demographic effects, but breeding probability, the probability that an individual will attempt to breed in a given year, remains one of the most difficult reproductive parameters to estimate reliably (Etterson et al., 2011). Individuals that do not breed are often more difficult or impossible to detect as they are not tied to a territory or breeding location. Therefore, to estimate breeding probability, we must account for variation in the sampling probabilities of breeders and nonbreeders (Nichols et al., 1994). In this study, we used a novel multievent‐robust design modeling approach to estimate survival and breeding probabilities of female elephant seals. Similar to other capture–recapture approaches exploiting multiple data sources (e.g., multistate models with dead recoveries and the Barker/robust design), a substantial advantage of our approach is that the nonbreeder state became “observable” when data collected during and outside of the breeding season were analyzed together. By accounting for uncertainty in the breeding status of individuals only seen outside of the breeding season, we could incorporate these auxiliary resightings within the flexible multievent framework. This allowed us to test for the existence of state‐dependent survival and to estimate breeding probability with greater precision.

Our results indicated that breeding probabilities subsequent to first reproduction best corresponded to a Markovian process, where breeders in year *t* tended to have a higher probability to breed again in the next year than females that were nonbreeders. On average, <20% of females skipped breeding after breeding in year *t* (i.e., *ψ* ^*B*−*B*^ > 0.80), a relatively low absolute rate of skipping compared to Weddell seal (*Leptonychotes weddellii*) (*ψ* ^*B*−*B*^ = 0.67, Chambert, Rotella, Higgs, & Garrott, 2013) and subantarctic fur seal (*Arctocephalus tropicalis*) females (*ψ* ^*B*−*B*^ = 0.59, Beauplet, Barbraud, Dabin, Küssener, & Guinet, 2006), respectively. Breeding probabilities at Marion Island were also higher and did not display as much temporal variation, as breeding probabilities of female elephant seals at Macquarie Island (Desprez, 2015). Nonbreeding females had lower subsequent breeding probabilities compared to breeding females at both Marion and Macquarie islands. Therefore, even though reproduction is energetically expensive in capital breeding elephant seals (sensu Jönsson, 1997), the energetic costs associated with breeding in a given year did not result in breeders being more likely to skip reproduction the year after compared to nonbreeders. This may suggest that factors other than reproductive costs—perhaps a high proportion of “frail” individuals entering the nonbreeder state in the first place—are important determinants of nonbreeding. If this is true, the mean state‐specific estimate of *ψ* ^*NB*−*B*^ may be adjusted upwards in models that account for “frail” individuals in the sample (Chambert et al., 2013). Such unobserved heterogeneity can be modeled with individual random effects in a multievent framework (Choquet & Gimenez, 2012; Gimenez & Choquet, 2010) or using the robust design approach in MARK (Stoelting, Gutiérrez, Kendall, & Peery, 2014). Alternatively, finite‐mixture models can be used to model discrete classes of heterogeneity (Pledger, Pollock, & Norris, 2003). Desprez, Gimenez, McMahon, Hindell, and Harcourt (2018) used this approach and found that “frail” female elephant seals had lower breeding probabilities and survival following breeding than females from the more robust heterogeneity class.

This study highlights that support for two pivotal assumptions that underpinned most previous work on breeding probability in southern elephant seals is unconvincing. Previous authors assumed constant detection or annual reproduction (Hindell, 1991; McMahon, Burton, & Bester, 2003) or interpreted capture probabilities obtained from Cormack–Jolly–Seber single state capture–recapture models (Pistorius & Bester, 2002; Pistorius, Bester, Hofmeyr, Kirkman, & Taylor, 2008; Pistorius et al., 2004), sometimes in combination with ad hoc methods (Pistorius, Bester, Kirkman, & Taylor, 2001), as the probability that a female elephant seal will breed in any year. There is clearly a high correlation between breeding probability and capture probability at the breeding colony, and given the generally high capture probability of breeding female elephant seals at Marion Island (Figure 5), we do not disregard the importance of these earlier findings. Still, these investigations were unable to formally separate breeding probabilities from capture probabilities, and their results are subject to some bias.

Models that describe the current population status and project future trajectories are highly relevant in evolutionary ecology and conservation biology, but require unbiased estimates of demographic parameters, including breeding probability. Biased estimates of breeding probability can thus undermine our ability to understand the processes that determine population growth rate (Kendall, Langtimm, Beck, & Runge, 2004). Our results are therefore relevant to the role that reproduction plays in the growth and viability of elephant seal populations (McMahon, Hindell, Burton, & Bester, 2005; Pistorius et al., 2001). Although annual survival affects the population growth rate of long‐lived animals disproportionately more than differences in annual breeding probability, breeding probability may drive population change because it tends to be more variable (Gaillard, Festa‐Bianchet, Yoccoz, Loison, & Toigo, 2000). Jenouvrier et al. (2005), for example, found that high temporal variation in breeding probability and breeding success had the strongest impact on the dynamics of a southern fulmar (*Fulmarus glacialoides*) population, even though the elasticity of population growth rate was high for adult survival and weak for breeding parameters. In accordance with the canalization hypothesis of life‐history traits (Gaillard & Yoccoz, 2003; Morris & Doak, 2004), our model selection results suggested stronger buffering in adult survival that consequently varied little between years, but that breeding probability fluctuated annually. This study aimed to derive a suitable analytic approach to estimate breeding probability and thus we did not additionally investigate whether environmental stochasticity (e.g., climate variation) may be driving the temporal variation in breeding probability. In iteroparous species, the proportion of individuals attempting to breed in a given year often correlates with environmental conditions (Paterson, Rotella, Arrigo, & Garrott, 2015; Van den Hoff et al., 2014). Although linkage between environmental variability and demographic parameters is desirable for the elephant seal population at Marion Island, it will command a better understanding of how changes in the physical environment propagate through the food web to influence the distribution and abundance of elephant seal prey.

### 4.1 Making use of multiple sampling occasions

Nonbreeders of many species (e.g., pond‐breeding amphibians, turtles, and colony‐breeding seabirds) routinely avoid breeding aggregations. Therefore, when observational sampling only occurs at breeding colonies, nonbreeders are often unobservable and can be considered to have temporarily emigrated from the study area. In such situations, multistate capture–recapture models can be used to estimate transition probabilities between observable and unobservable states (Fujiwara & Caswell, 2002; Kendall & Nichols, 2002). These models perform well when temporary emigration is Markovian, as transition probabilities to and from unobservable nonbreeding states frequently are (Schaub, Gimenez, Schmidt, & Pradel, 2004). Unobservable states nonetheless cause parameter redundancy problems in estimation, especially in models with complex transition probability structures. Furthermore, state‐dependent survival cannot be estimated if nonbreeders occupy an unobservable state as their fate cannot be directly monitored (Kendall & Bjorkland, 2001). Consequently, there is no information in the likelihood function to distinguish whether survival differed between observable and unobservable individuals. The assumption of equal survival among breeders and nonbreeders is a severe limitation, as the difference in survival probability of breeders and nonbreeders is often a key interest. In this study, for example, we found some evidence for lower survival in breeders compared to nonbreeders, perhaps attributed to the survival cost incurred by first‐time breeders in particular (Desprez et al., 2014). The best solution to deal with unobservable life‐history states is therefore to eliminate them altogether (Kendall, 2004). This may not always be possible, but sampling designs that include survey effort away from the breeding colony, or continue to search for marked individuals outside of the breeding season, may recapture sufficient numbers of individuals which would otherwise be unobservable.

Unobservable life‐history states may be eliminated, parameter precision be increased and parameter redundancy reduced using multistate models with multiple underlying data sources. Multiple data sources may refer to robust design sampling (Kendall, Nichols, & Hines, 1997), dead recoveries (Catchpole, Freeman, Morgan, & Harris, 1998), auxiliary (or incidental) resightings (Barker, 1997), satellite telemetry (Kendall et al., 2009), or combinations thereof (Kendall, White, Hines, Langtimm, & Yoshizaki, 2012; Lindberg et al., 2001). The Barker/robust design (Kendall et al., 2013), for example, maximizes the precision of parameter estimates by combining robust design sampling, recoveries, and auxiliary resightings. Robust design in combination with auxiliary resightings of live individuals outside of the primary sampling area or period is a very effective way of reducing terminal bias in survival estimates under Markovian temporary emigration (Peñaloza, Kendall, & Langtimm, 2014). Despite the benefits of increased precision of parameter estimates and user‐friendly implementation in program MARK (White & Burnham, 1999), the Barker/robust design approach has only rarely been used in empirical applications to estimate survival and population size or density (as derived parameters) of wild animal populations (Fabrizio, Tuckey, Latour, White, & Norris, 2018; Gómez‐Ramírez, Gutiérrez‐González, & López‐González, 2017; Gutiérrez‐González, Gómez‐Ramírez, López‐González, & Doherty, 2015; Ivan, White, & Shenk, 2014; Weithman et al., 2017). Weithman et al. (2017) additionally interpreted the “availability” parameter (*a*ʹ, the probability of being available for capture given that the individual was previously unavailable for capture) as a measure of breeding probability in piping plover (*Charadrius melodus*), a migratory shorebird. The Barker/robust design implemented in program MARK currently lacks multistate and/or multievent extensions, but the multistate extension of the Barker model (Barker, 1997) allows an alternative, direct means of estimating breeding probability and accommodates uncertain identification of states. This model, however, foregoes the advantage of a robust design structure within primary periods.

The multievent‐robust design model we used to estimate breeding probability in elephant seals is similar to the Barker/robust design (Kendall et al., 2013) in that it combined robust design data collected during discrete breeding seasons with observations made at other times of the year. Within each breeding season, the encounter history matrix of the multievent‐robust design model summarized *j* = 8 weekly secondary occasions as two capture periods. Because the staggered arrival and departure of breeding female elephant seals violates the assumption of geographic closure within a breeding season, we collapsed alternating secondary occasions (e.g., *j* = {1, 3, 5, 7}) to generate two distinct capture periods per breeding season. Although this data aggregation involves some information loss, we found that the two capture periods summarized sufficient information in breeding season capture probabilities to correctly discern state uncertainty. In this study, we always allowed capture probabilities to differ between “uneven” and “even” capture periods (*κ* ^*U*^ and *κ* ^*E*^), but these can also be constrained to be the same by aggregating parameters across the event matrices using the *Set equality between parameters of various types* function in E‐SURGE.

Although we did not consider the prebreeder stage of the life cycle in this study, it is straightforward to include observations of prebreeders using the multievent‐robust design framework (Supporting Information Appendix S4). Breeding recruitment (the probability to breed for the first time) can then be estimated as the transition from prebreeder to breeder. Similarly, we did not include dead recoveries, but such extension would not be difficult to incorporate if dead recovery data are available. By combining recapture data from the breeding season and the moult, we effectively assume no mortality occurs between breeding and moult. The main consequence of violation of this assumption is that detection during the moult tends to be underestimated (individuals that have died since breeding are considered as alive and missed, whereas they are not present). Mortality, on the other hand, is simply recorded with a delay (i.e., in the interval between the moult and next year breeding season), with no consequence for the survival parameter, which corresponds to an estimate of annual survival.

In general, multievent models (typically implemented in program E‐SURGE, Choquet et al., 2009) provide a flexible means to model multiple data sources. For example, Sanz‐Aguilar et al. (2011) combined nest‐based information with capture–recapture data to estimate breeding probability in Cory's Shearwater (*Calonectris diomedea*). In another application, Souchay, Gauthier, and Pradel (2014) combined resights of greater snow geese (*Chen caerulescens atlantica*) during the nesting stage with recaptures at the end of the breeding season to develop a robust design‐like model that can be used to study breeding probability within a multievent framework. Cayuela et al. (2014, 2017) recaptured yellow‐bellied toads (*Bombina variegata*) during multiple field sessions in a year and used multievent models with multiple secondary capture sessions to investigate how environmental variation influences breeding decisions in this species. Finally, Richard, Toïgo, Appolinaire, Loison, and Garel (2017) integrated robust design in a multievent framework to distinguish reproductive costs related to variable energy allocation from gestation to weaning in Pyrenean chamois (*Rupicapra pyrenaica pyrenaica*). Although these studies (as well as our own study) were all based on incorporating multiple data sources in a multievent framework, the general model structure differed in every study. Indeed, a key advantage of multievent models is seated in their flexibility to incorporate auxiliary information arising from a diverse array of imperfect observations. Although the joint analysis of multiple data sources improves parameter estimation, the level of model complexity grows with the inclusion of several sources of data. A potential concern for multievent models with state uncertainty is that they are prone to local minima during the likelihood maximization routine (Choquet et al., 2009). Care should thus be taken to ensure that models converge to the lowest deviance. We used the Expectation Maximization (EM) algorithm combined with Quasi‐Newton minimization methods implemented in E‐SURGE and ran the same models multiple times using different randomly chosen starting values to reduce the risk of convergence to a local minima.

A range of advanced capture–recapture model‐fitting approaches is available to ecologists for estimation of breeding probability and other demographic parameters. We initially encountered limitations with prevailing approaches, and thus endeavored to estimate breeding probability using a multievent‐robust design approach. For example, we found considerable positive bias in estimates of breeding probability in multievent models fitted without the benefit of a robust design structure in the breeding season (i.e., when all breeding survey data were aggregated to one [instead of two] capture periods) (Oosthuizen, 2016). In this model, all individuals observed only outside of the breeding season were also assigned to an “uncertain” event, and the model considered the possibility that either the individual was a nonbreeder, or the individual was present and breeding, but escaped detection. However, without a robust design structure during the breeding season, the multievent model appeared to assign females seen with an “uncertain” event to the breeder state even in years with apparent “perfect” breeder detection probabilities (Supporting Information Appendix S5). Desprez et al. (2018) used a different multievent design with state uncertainty to study intermittent breeding in elephant seals at Macquarie Island, but their results also indicated maximum‐likelihood estimation problems in the absence of robust design. Breeding state assignment probabilities were estimated on the boundary of parameter space (i.e., when a parameter is estimated as 0 or 1), suggestive of *p* = 1 during the breeding season, which is suspicious for that population. We also considered analyzing our data using a multistate open robust design (MSORD) model (Kendall & Bjorkland, 2001) (Supporting Information Appendix S6). In this case, surveys conducted outside of the breeding season did not contribute data to the analysis and therefore the nonbreeder state was unobservable. As such, the MSORD analysis could not estimate state‐dependent survival. However, in some studies, this disadvantage may be offset by a specific interest in the derived parameters from a MSORD analysis (population size, residence time, and the effective capture probability) (Supporting Information Appendix S7).

## 5 CONCLUSION

The trade‐offs that exist between annual and intermittent breeding play a central role in life‐history theory (Stearns, 1989). In limiting conditions, long‐lived animals are inclined to trade annual reproduction for improved survival prospects; consequently, breeding probability is one of the demographic rates that vary most along with temporal fluctuations in environmental conditions (Gaillard et al., 2000; Pfister, 1998). In an increasingly variable world, environmental stochasticity should intensify temporal variance in breeding probabilities, and heighten among‐individual differences in reproductive trajectories. These changes will have varied, but often negative effects on individual fitness and population growth (Doak, Morris, Pfister, Kendall, & Bruna, 2005), and are therefore important to monitor.

Capture–recapture studies which make use of multiple data sources offer powerful approaches to study breeding probability and other demographic parameters. Sampling protocols which include multiple data sources have the potential to produce inferences that are less biased and more precise, given that investigators utilize these multiple data sources during analysis (Kendall et al., 2013). The multievent‐robust design approach we illustrated can easily be adapted for any state process. The observation process is flexible and can be adapted to suit field data from numerous species which may be encountered during and outside of discrete breeding seasons. We encourage ecologists and wildlife managers to collect and utilize robust design data to ensure the highest‐quality inference from their data. We further advocate that, when available, observations of nonbreeding individuals should be incorporated in analysis, as this can significantly improve the precision and accuracy of demographic parameter estimates (Kendall, 2004; Pardo, Weimerskirch, & Barbraud, 2013).

## CONFLICT OF INTEREST

None declared.

## AUTHOR CONTRIBUTIONS

WCO and RP formulated the idea. MNB and PJNdB coordinated elephant seal field work and provided data. WCO analyzed the data and wrote the manuscript with input from all authors.

## Data Availability

Data available from the Dryad Digital Repository: https://doi.org/10.5061/dryad.gd23252.

## References

[ece34828-bib-0001] Barbraud, C. , & Weimerskirch, H. (2012). Estimating survival and reproduction in a quasi‐biennially breeding seabird with uncertain and unobservable states. Journal of Ornithology, 152, S605–S615. 10.1007/s10336-011-0686-1

[ece34828-bib-0002] Barker, R. J. (1997). Joint modeling of live‐recapture, tag‐resight, and tag‐recovery data. Biometrics, 53, 666–677. 10.2307/2533966

[ece34828-bib-0003] Barker, R. J. , Burnham, K. P. , & White, G. C. (2004). Encounter history modeling of joint mark recapture, tag‐resighting and tag‐recovery data under temporary emigration. Statistica Sinica, 14, 1037–1055.

[ece34828-bib-0004] Barker, R. J. , & White, G. C. (2004). Towards the mother‐of‐all‐models: Customised construction of the mark–recapture likelihood function. Animal Biodiversity and Conservation, 27, 177–185.

[ece34828-bib-0005] Baron, J.‐P. , Le Galliard, J.‐F. , Ferrière, R. , & Tully, T. (2013). Intermittent breeding and the dynamics of resource allocation to reproduction, growth and survival. Functional Ecology, 27, 173–183. 10.1111/1365-2435.12023

[ece34828-bib-0006] Beauplet, G. , Barbraud, C. , Dabin, W. , Küssener, C. , & Guinet, C. (2006). Age‐specific survival and reproductive performances in fur seals: Evidence of senescence and individual quality. Oikos, 112, 430–441. 10.1111/j.0030-1299.2006.14412.x

[ece34828-bib-0007] Bull, J. J. , & Shine, R. (1979). Iteroparous animals that skip opportunities for reproduction. American Naturalist, 114, 296–303. 10.1086/283476

[ece34828-bib-0008] Burnham, K. P. , & Anderson, D. R. (2002). Model selection and multimodel inference. A practical information‐theoretic approach (2nd ed.). New York, NY: Springer Science and Business Media Inc..

[ece34828-bib-0009] Cam, E. , Hines, J. E. , Monnat, J.‐Y. , Nichols, J. D. , & Danchin, E. (1998). Are adult nonbreeders prudent parents? The kittiwake model. Ecology, 79, 2917–2930. 10.1890/0012-9658(1998)079[2917:AANPPT]2.0.CO;2

[ece34828-bib-0010] Catchpole, E. A. , Freeman, S. N. , Morgan, B. J. T. , & Harris, M. P. (1998). Integrated recovery/recapture data analysis. Biometrics, 54, 33–46. 10.2307/2533993

[ece34828-bib-0011] Cayuela, H. , Besnard, A. , Bonnaire, E. , Perret, H. , Rivoalen, J. , Miaud, C. , & Joly, P. (2014). To breed or not to breed: Past reproductive status and environmental cues drive current breeding decisions in a long‐lived amphibian. Oecologia, 176, 107–116. 10.1007/s00442-014-3003-x 24996543

[ece34828-bib-0012] Cayuela, H. , Joly, P. , Schmidt, B. R. , Pichenot, J. , Bonnaire, E. , Priol, P. , … Besnard, A. (2017). Life history tactics shape amphibians’ demographic responses to the North Atlantic Oscillation. Global Change Biology, 23, 4620–4638. 10.1111/gcb.13672 28236653

[ece34828-bib-0013] Chambert, T. , Rotella, J. J. , Higgs, M. D. , & Garrott, R. A. (2013). Individual heterogeneity in reproductive rates and cost of reproduction in a long‐lived vertebrate. Ecology and Evolution, 3, 2047–2060. 10.1002/ece3.615 23919151 PMC3728946

[ece34828-bib-0014] Choquet, R. , & Gimenez, O. (2012). Towards built‐in capture‐recapture mixed models in program E‐SURGE. Journal of Ornithology, 152, S625–S639. 10.1007/s10336-010-0613-x

[ece34828-bib-0015] Choquet, R. , Rouan, L. , & Pradel, R. (2009). Program E‐SURGE: A software application for fitting multievent models In Thomson D. L., Cooch E. G., & Conroy M. J. (Eds.), Modeling demographic processes in marked populations (pp. 845–865). New York, NY: Springer 10.1007/978-0-387-78151-8

[ece34828-bib-0016] Church, D. R. , Bailey, L. L. , Wilbur, H. M. , Kendall, W. L. , & Hines, J. E. (2007). Iteroparity in the variable environment of the salamander *Ambystoma tigrinum* . Ecology, 88, 891–903. 10.1890/06-0896 17536706

[ece34828-bib-0017] Cohen, J. B. , Maddock, S. B. , Bimbi, M. K. , Golder, W. W. , Ledee, O. E. , Cuthbert, F. J. , … Gratto‐Trevor, C. L. (2018). State uncertainty models and mark‐resight models for understanding nonbreeding site use by Piping Plovers. Ibis, 160, 342–354. 10.1111/ibi.12546

[ece34828-bib-0018] Converse, S. J. , Kendall, W. L. , Doherty, P. F. , & Ryan, P. G. (2009). Multistate models for estimation of survival and reproduction in the grey‐headed albatross (*Thalassarche chrysostoma*). Auk, 126, 77–88. 10.1525/auk.2009.07189

[ece34828-bib-0019] De Bruyn, P. J. N. , Tosh, C. A. , Bester, M. N. , Cameron, E. Z. , McIntyre, T. , & Wilkinson, I. S. (2011). Sex at sea: Alternative mating system in an extremely polygynous mammal. Animal Behaviour, 82, 445–451. 10.1016/j.anbehav.2011.06.006

[ece34828-bib-0020] Desprez, M. (2015). Southern Ocean sentinels: Demographic insights into the declining population of southern elephant seals at Macquarie Island. PhD thesis, Macquarie University, Sydney, NSW.

[ece34828-bib-0021] Desprez, M. , Gimenez, O. , McMahon, C. R. , Hindell, M. A. , & Harcourt, R. G. (2018). Optimizing lifetime reproductive output: intermittent breeding as a tactic for females in a long‐lived, multiparous mammal. Journal of Animal Ecology, 87, 199–211. 10.1111/1365-2656.12775 29063588

[ece34828-bib-0022] Desprez, M. , Harcourt, R. , Hindell, M. A. , Cubaynes, S. , Gimenez, O. , & McMahon, C. R. (2014). Age‐specific cost of first reproduction in female southern elephant seals. Biology Letters, 10, 20140264 10.1098/rsbl.2014.0264 24872464 PMC4046382

[ece34828-bib-0023] Doak, D. F. , Morris, W. F. , Pfister, C. , Kendall, B. E. , & Bruna, E. M. (2005). Correctly estimating how environmental stochasticity influences fitness and population growth. American Naturalist, 166, E14–E21. 10.1086/430642 15937784

[ece34828-bib-0024] Drent, R. H. , & Daan, S. (1980). The prudent parent: Adjustments in avian breeding. Ardea, 68, 225–252.

[ece34828-bib-0025] Etterson, M. A. , Ellis‐Felege, S. N. , Evers, D. , Gauthier, G. , Grzybowski, J. A. , Mattsson, B. J. , … Potvien, A. (2011). Modeling fecundity in birds: Conceptual overview, current models, and considerations for future developments. Ecological Modelling, 222, 2178–2190. 10.1016/j.ecolmodel.2010.10.013

[ece34828-bib-0026] Fabrizio, M. C. , Tuckey, T. D. , Latour, R. J. , White, G. C. , & Norris, A. J. (2018). Tidal habitats support large numbers of invasive blue catfish in a Chesapeake Bay Subestuary. Estuaries and Coasts, 41, 827–840.

[ece34828-bib-0027] Fujiwara, M. , & Caswell, H. (2002). A general approach to temporary emigration in mark‐recapture analysis. Ecology, 83, 3266–3275.

[ece34828-bib-0028] Gaillard, J. M. , Festa‐Bianchet, M. , Yoccoz, N. G. , Loison, A. , & Toigo, C. (2000). Temporal variation in fitness components and population dynamics of large herbivores. Annual Review of Ecology and Systematics, 31, 367–393. 10.1146/annurev.ecolsys.31.1.367

[ece34828-bib-0029] Gaillard, J. M. , & Yoccoz, N. G. (2003). Temporal variation in survival of mammals: A case of environmental canalization? Ecology, 84, 3294–3306. 10.1890/02-0409

[ece34828-bib-0030] Gimenez, O. , & Choquet, R. (2010). Incorporating individual heterogeneity in studies on marked animals using numerical integration: Capture‐recapture mixed models. Ecology, 91, 951–957. 10.1890/09-1903.1 20462110

[ece34828-bib-0031] Gimenez, O. , Lebreton, J. D. , Gaillard, J. M. , Choquet, R. , & Pradel, R. (2012). Estimating demographic parameters using hidden process dynamic models. Theoretical Population Biology, 82, 307–316. 10.1016/j.tpb.2012.02.001 22373775

[ece34828-bib-0032] Gómez‐Ramírez, M. A. , Gutiérrez‐González, C. E. , & López‐González, C. A. (2017). Ocelots thrive in a non‐typical habitat of northwestern Mexico. Endangered Species Research, 32, 471–478. 10.3354/esr00828

[ece34828-bib-0033] Gutiérrez‐González, C. E. , Gómez‐Ramírez, M. A. , López‐González, C. A. , & Doherty, P. F. Jr (2015). Are private reserves effective for jaguar conservation? PLoS ONE, 10, e0137541 10.1371/journal.pone.0137541 26398115 PMC4580466

[ece34828-bib-0034] Hindell, M. A. (1991). Some life‐history parameters of a declining population of southern elephant seals, *Mirounga leonina* . Journal of Animal Ecology, 60, 119–134. 10.2307/5449

[ece34828-bib-0035] Hindell, M. A. , & Burton, H. R. (1988). Seasonal haul‐out patterns of the southern elephant seal (*Mirounga leonina* L.), at Macquarie Island. Journal of Mammalogy, 69, 81–88. 10.2307/1381750

[ece34828-bib-0036] Ivan, J. S. , White, G. C. , & Shenk, T. M. (2014). Density and demography of snowshoe hares in Central Colorado. Journal of Wildlife Management, 78, 580–594. 10.1002/jwmg.695

[ece34828-bib-0037] Jenouvrier, S. , Barbraud, C. , Cazelles, B. , & Weimerskirch, H. (2005). Modelling population dynamics of seabirds: Importance of the effects of climate fluctuations on breeding proportions. Oikos, 108, 511–522. 10.1111/j.0030-1299.2005.13351.x

[ece34828-bib-0038] Jönsson, K. I. (1997). Capital and income breeding as alternative tactics of resource use in reproduction. Oikos, 78, 57–66. 10.2307/3545800

[ece34828-bib-0039] Jørgensen, C. , Ernande, B. , Fiksen, Ø. , & Dieckmann, U. (2006). The logic of skipped spawning in fish. Canadian Journal of Fisheries and Aquatic Sciences, 63, 200–211. 10.1139/f05-210

[ece34828-bib-0040] Kendall, W. L. (2004). Coping with unobservable and mis–classified states in capture–recapture studies. Animal Biodiversity and Conservation, 27, 97–107.

[ece34828-bib-0041] Kendall, W. L. , Barker, R. J. , White, G. C. , Lindberg, M. S. , Langtimm, C. A. , & Peñaloza, C. L. (2013). Combining dead recovery, auxiliary observations and robust design data to estimate demographic parameters from marked individuals. Methods in Ecology and Evolution, 4, 828–835. 10.1111/2041-210X.12077

[ece34828-bib-0042] Kendall, W. L. , & Bjorkland, R. (2001). Using open robust design models to estimate temporary emigration from capture–recapture data. Biometrics, 57, 1113–1122. 10.1111/j.0006-341X.2001.01113.x 11764251

[ece34828-bib-0043] Kendall, W. L. , Converse, S. J. , Doherty, P. F. Jr , Naughton, M. B. , Anders, A. , Hines, J. E. , & Flint, E. (2009). Sampling design considerations for demographic studies: A case of colonial seabirds. Ecological Applications, 19, 55–68. 10.1890/07-1072.1 19323173

[ece34828-bib-0044] Kendall, W. L. , Hines, J. E. , & Nichols, J. D. (2003). Adjusting multistate capture‐recapture models for misclassification bias: Manatee breeding proportions. Ecology, 84, 1058–1066. 10.1890/0012-9658(2003)084[1058:AMCMFM]2.0.CO;2

[ece34828-bib-0045] Kendall, W. L. , Langtimm, C. A. , Beck, C. A. , & Runge, M. C. (2004). Capture‐recapture analysis for estimating manatee reproductive rates. Marine Mammal Science, 20, 424–437. 10.1111/j.1748-7692.2004.tb01170.x

[ece34828-bib-0046] Kendall, W. L. , & Nichols, J. D. (1995). On the use of secondary capture‐recapture samples to estimate temporary emigration and breeding proportions. Journal of Applied Statistics, 22, 751–762. 10.1080/02664769524595

[ece34828-bib-0047] Kendall, W. L. , & Nichols, J. D. (2002). Estimating state‐transition probabilities for unobservable states using capture‐recapture/resighting data. Ecology, 83, 3276–3284.

[ece34828-bib-0048] Kendall, W. L. , Nichols, J. D. , & Hines, J. E. (1997). Estimating temporary emigration using capture–recapture data with Pollock's robust design. Ecology, 78, 563–578.

[ece34828-bib-0049] Kendall, W. L. , White, G. C. , Hines, J. E. , Langtimm, C. A. , & Yoshizaki, J. (2012). Estimating parameters of hidden Markov models based on marked individuals: Use of robust design data. Ecology, 93, 913–920. 10.1890/11-1538.1 22690641

[ece34828-bib-0050] Kirkman, S. P. , Bester, M. N. , Pistorius, P. A. , Hofmeyr, G. J. G. , Jonker, F. C. , Owen, R. , & Strydom, N. (2003). Variation in the timing of moult in southern elephant seals at Marion Island. South African Journal of Wildlife Research, 33, 79–84.

[ece34828-bib-0051] Kirkman, S. P. , Bester, M. N. , Pistorius, P. A. , Hofmeyr, G. J. G. , Owen, R. , & Mecenero, S. (2001). Participation in the winter haulout by southern elephant seals (*Mirounga leonina*). Antarctic Science, 13, 380–384.

[ece34828-bib-0052] Le Boeuf, B. J. , & Laws, R. M. (1994). Elephant seals: An introduction to the genus In Le Boeuf B. J., & Laws R. M. (Eds.), Elephant seals: Population ecology, behavior, and physiology (pp. 1–26). Berkeley, CA: University of California Press.

[ece34828-bib-0053] Lebreton, J. D. , Nichols, J. D. , Barker, R. J. , Pradel, R. , & Spendelow, J. A. (2009). Modeling individual animal histories with multistate capture‐recapture models. Advances in Ecological Research, 41, 87–173. 10.1016/S0065-2504(09)00403-6

[ece34828-bib-0054] Lebreton, J. D. , & Pradel, R. (2002). Multistate recapture models: Modeling incomplete individual histories. Journal of Applied Statistics, 29, 353–369. 10.1080/02664760120108638

[ece34828-bib-0055] Lindberg, M. S. , Kendall, W. L. , Hines, J. E. , & Anderson, M. G. (2001). Combining band recovery data and Pollock's robust design to model temporary and permanent emigration. Biometrics, 57, 273–282. 10.1111/j.0006-341X.2001.00273.x 11252610

[ece34828-bib-0056] McMahon, C. R. , Burton, H. R. , & Bester, M. N. (2003). A demographic comparison of two southern elephant seal populations. Journal of Animal Ecology, 72, 61–74. 10.1046/j.1365-2656.2003.00685.x

[ece34828-bib-0057] McMahon, C. R. , Hindell, M. A. , Burton, H. R. , & Bester, M. N. (2005). Comparison of southern elephant seal populations, and observations of a population on a demographic knife‐edge. Marine Ecology Progress Series, 288, 273–283. 10.3354/meps288273

[ece34828-bib-0058] Morris, W. F. , & Doak, D. F. (2004). Buffering of life histories against environmental stochasticity: Accounting for a spurious correlation between the variabilities of vital rates and their contributions to fitness. American Naturalist, 163, 579–590. 10.1086/382550 15122504

[ece34828-bib-0059] Muths, E. , Scherer, R. D. , & Lambert, B. A. (2010). Unbiased survival estimates and evidence for skipped breeding opportunities in females. Methods in Ecology and Evolution, 1, 123–130. 10.1111/j.2041-210X.2010.00019.x

[ece34828-bib-0060] Nichols, J. D. , Hines, J. E. , Pollock, K. H. , Hinz, R. L. , & Link, W. A. (1994). Estimating breeding proportions and testing hypotheses about costs of reproduction with capture‐recapture data. Ecology, 75, 2052–2065. 10.2307/1941610

[ece34828-bib-0061] Oosthuizen, W. C. (2016). Life history and demographic consequences of individual heterogeneity in southern elephant seals. PhD thesis, University of Pretoria, Pretoria, South Africa.

[ece34828-bib-0062] Oosthuizen, W. C. , Altwegg, R. , Nevoux, M. , Bester, M. N. , & de Bruyn, P. J. N. (2018). Phenotypic selection and covariation in the life‐history traits of elephant seals: Heavier offspring gain a double selective advantage. Oikos, 127, 875–889. 10.1111/oik.04998

[ece34828-bib-0063] Pardo, D. , Weimerskirch, H. , & Barbraud, C. (2013). When celibacy matters: Incorporating nonbreeders improves demographic parameter estimates. PLoS ONE, 8, e60389 10.1371/journal.pone.0060389 23555965 PMC3612038

[ece34828-bib-0064] Paterson, J. T. , Rotella, J. J. , Arrigo, K. R. , & Garrott, R. A. (2015). Tight coupling of primary production and marine mammal reproduction in the Southern Ocean. Proceedings of the Royal Society B, 282, 20143137 10.1098/rspb.2014.3137 25854885 PMC4426618

[ece34828-bib-0065] Peñaloza, C. L. , Kendall, W. L. , & Langtimm, C. A. (2014). Reducing bias in survival under non‐random temporary emigration. Ecological Applications, 24, 1155–1166. 10.1890/13-0558.1 25154103

[ece34828-bib-0066] Pfister, C. A. (1998). Patterns of variance in stage‐structured populations: Evolutionary predictions and ecological implications. Proceedings of the National Academy of Sciences, USA, 95, 213–218. 10.1073/pnas.95.1.213 PMC34554 9419355

[ece34828-bib-0067] Pilastro, A. , Tavecchia, G. , & Marin, G. (2003). Long living and reproduction skipping in the fat dormouse. Ecology, 84, 1784–1792. 10.1890/0012-9658(2003)084[1784:LLARSI]2.0.CO;2

[ece34828-bib-0068] Pistorius, P. A. , & Bester, M. N. (2002). A longitudinal study of senescence in a pinniped. Canadian Journal of Zoology, 80, 395–401. 10.1139/z02-017

[ece34828-bib-0069] Pistorius, P. A. , Bester, M. N. , Hofmeyr, G. J. G. , Kirkman, S. P. , & Taylor, F. E. (2008). Seasonal survival and the relative cost of first reproduction in adult female southern elephant seals. Journal of Mammalogy, 89, 567–574. 10.1644/07-MAMM-A-219R.1

[ece34828-bib-0070] Pistorius, P. A. , Bester, M. N. , Kirkman, S. P. , & Taylor, F. E. (2001). Temporal changes in fecundity and age at sexual maturity of southern elephant seals at Marion Island. Polar Biology, 24, 343–348. 10.1007/s003000000217

[ece34828-bib-0071] Pistorius, P. A. , Bester, M. N. , Lewis, M. N. , Taylor, F. E. , Campagna, C. , & Kirkman, S. P. (2004). Adult female survival, population trend, and the implications of early primiparity in a capital breeder, the southern elephant seal (*Mirounga leonina*). Journal of Zoology, 263, 107–119. 10.1017/S0952836904004984

[ece34828-bib-0072] Pistorius, P. A. , de Bruyn, P. J. N. , & Bester, M. N. (2011). Population dynamics of southern elephant seals: A synthesis of three decades of demographic research at Marion Island. African Journal of Marine Science, 33, 523–534. 10.2989/1814232X.2011.637357

[ece34828-bib-0073] Pledger, S. , Pollock, K. H. , & Norris, J. L. (2003). Open capture‐recapture models with heterogeneity: I. Cormack–Jolly–Seber model. Biometrics, 59, 786–794. 10.1111/j.0006-341X.2003.00092.x 14969456

[ece34828-bib-0074] Pollock, K. H. (1982). A capture‐recapture design robust to unequal probability of capture. Journal of Wildlife Management, 46, 757–760.

[ece34828-bib-0075] Pradel, R. (2005). Multievent: An extension of multistate capture recapture models to uncertain states. Biometrics, 61, 442–447. 10.1111/j.1541-0420.2005.00318.x 16011690

[ece34828-bib-0076] Pradel, R. (2009). The stakes of capture–recapture models with state uncertainty In Thomson D. L., Cooch E. G. & Conroy M. J. (Eds.), Modeling demographic processes in marked populations. Environmental and ecological statistics (pp. 781–796). New York, NY: Springer.

[ece34828-bib-0077] Reed, T. E. , Harris, M. P. , & Wanless, S. (2015). Skipped breeding in common guillemots in a changing climate: Restraint or constraint? Frontiers in Ecology and Evolution, 3, 1 10.3389/fevo.2015.00001

[ece34828-bib-0078] Richard, Q. , Toïgo, C. , Appolinaire, J. , Loison, A. , & Garel, M. (2017). From gestation to weaning: Combining robust design and multi‐event models unveils cost of lactation in a large herbivore. Journal of Animal Ecology, 86, 1497–1509. 10.1111/1365-2656.12736 28772345

[ece34828-bib-0079] Rivalan, P. , Prévot‐Julliard, A.‐C. , Choquet, R. , Pradel, R. , Jacquemin, B. , & Girondot, M. (2005). Trade‐off between current reproductive effort and delay to next reproduction in the leatherback sea turtle. Oecologia, 145, 564–574. 10.1007/s00442-005-0159-4 16028096

[ece34828-bib-0080] Ruiz‐Gutierrez, V. , Kendall, W. L. , Saracco, J. F. , & White, G. C. (2016). Overwintering strategies of migratory birds: A novel approach for estimating seasonal movement patterns of residents and transients. Journal of Applied Ecology, 53, 1035–1045. 10.1111/1365-2664.12655

[ece34828-bib-0081] Sanz‐Aguilar, A. , Tavecchia, G. , Genovart, M. , Manuel Igual, J. , Oro, D. , Rouan, L. , & Pradel, R. (2011). Studying the reproductive skipping behavior in long‐lived birds by adding nest inspection to individual‐based data. Ecological Applications, 21, 555–564. 10.1890/09-2339.1 21563585

[ece34828-bib-0082] Schaub, M. , Gimenez, O. , Schmidt, B. R. , & Pradel, R. (2004). Estimating survival and temporary emigration in the multistate capture–recapture framework. Ecology, 85, 2107–2113. 10.1890/03-3110

[ece34828-bib-0083] Souchay, G. , Gauthier, G. , & Pradel, R. (2014). To breed or not: A novel approach to estimate breeding propensity and potential trade‐offs in an Arctic‐nesting species. Ecology, 95, 2745–2756. 10.1890/13-1277.1

[ece34828-bib-0084] Stearns, S. C. (1989). Trade‐offs in life‐history evolution. Functional Ecology, 3, 259–268. 10.2307/2389364

[ece34828-bib-0085] Stoelting, R. E. , Gutiérrez, R. J. , Kendall, W. L. , & Peery, M. Z. (2014). Life‐history tradeoffs and reproductive cycles in Spotted Owls. The Auk, 132, 46–64.

[ece34828-bib-0086] van den Hoff, J. , McMahon, C. R. , Simpkins, G. R. , Hindell, M. A. , Alderman, R. , & Burton, H. R. (2014). Bottom‐up regulation of a pole‐ward migratory predator population. Proceedings of the Royal Society B, 281, 20132842 10.1098/rspb.2013.2842 24619437 PMC3973255

[ece34828-bib-0087] Weithman, C. , Gibson, D. , Hunt, K. , Friedrich, M. , Fraser, J. , Karpanty, S. , & Catlin, D. (2017). Senescence and carryover effects of reproductive performance influence migration, condition, and breeding propensity in a small shorebird. Ecology and Evolution, 7, 11044–11056. 10.1002/ece3.3533 29299280 PMC5743479

[ece34828-bib-0088] White, G. C. , & Burnham, K. P. (1999). Program MARK: Survival estimation from populations of marked animals. Bird Study, 46, S120–S139. 10.1080/00063659909477239

