# Supplementary material for: Making use of multiple surveys: Estimating breeding probability using a multievent‐robust design capture–recapture model
Source: Ecol Evol. 2019 Feb 5;9(2):836–48. doi: 10.1002/ece3.4828 (PMC6362610; doi:10.1002/ece3.4828)
Supplement: Supplementary file 1 [file ECE3-9-836-s001.docx]

**Supporting Information S1**


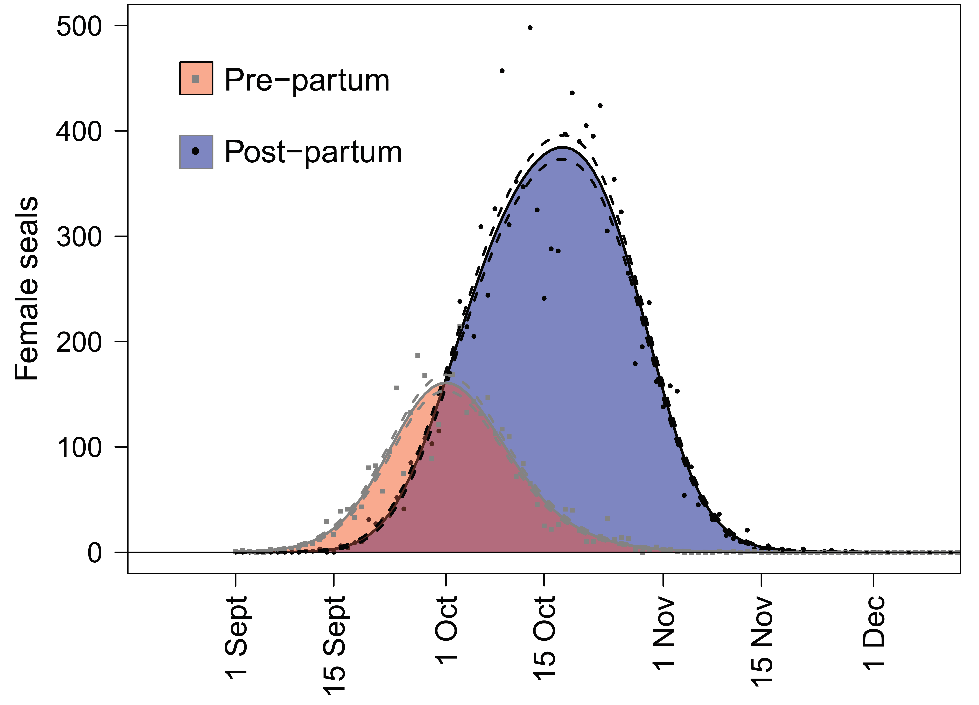


**Supporting Information S1: Fig. S1.** The seasonal distribution of female elephant seals in harems at Marion Island during breeding seasons. Pre-partum (pregnant) and post-partum (lactating) females are represented by grey squares (pink shading [the first curve]) and black circles (blue shading [taller, second curve]). The x-axis corresponds to date.

**Supporting Information S1**

**Supporting Information S1: Fig. S2.** The average number of tagged female southern elephant seals observed per day at Marion Island (1983 to 2013). Panel A. Weaned pups (green shading [tall curve]) are repeatedly observed ashore during the post-weaning fast, and again as under-yearlings (pink shading [second curve]) during the winter of the following year. Panels B and C. Age 1 pre-breeders (yearlings) and age 2 pre-breeders (sub-adults) are observed during moult (~ Nov to Jan) and winter haulouts. Panel D. Age 3 females may start to breed at which time they become ‘adult females’ (~Sept – Nov). Those age 3 females that do not breed are at sea during the breeding season, and moult earlier than age 3 breeders. Very few female seals age 3+ are subsequently observed during the winter. The x-axis corresponds to a ‘seal year’, i.e., the annual cycle from September to August.

**Supporting Information S1**

**Supporting Information S1: Fig. S3.** The timing and duration of the breeding and moult haulout of female elephant seals at Marion Island. Adult females (i.e., ‘breeders’ - individuals that have bred at least once before) are compared to pre-breeders (individuals not known to have bred). The moult haulout of age 3 pre-breeders clearly precedes that of same-aged breeders.

**Supporting Information S2 *– Including non-breeders and state uncertainty in the breeding season robust design***

Non-breeding southern elephant seals avoid breeding colonies most of the time (de Bruyn et al. 2011). The multievent-robust design model described in the main text assumed that any female observed ashore during a secondary sampling period was a breeder. Field observations during the breeding season distinguish between sightings of females with pups and those seen without pups in close proximity. A small number of females ashore in the breeding season (typically five to 10 individuals, representing < 2% of the breeding population at Marion Island) are never seen with pups in close proximity. Such females may be non-breeders attending the breeding colony (to mate, for example). Alternatively, females only observed without pups may be breeders which have only been observed pre-partum, or after the death (pre-weaning mortality of pups is < 5%, Pistorius et al. 2001) or weaning of their pups. Pups may also elude detection if they are temporarily separated from their mothers.

Uncertainty about the true breeding status of a small number of females present in the breeding season thus exists. Given that so few females are never seen without a pup, all females observed ashore during the breeding season are usually assigned to the breeder state (e.g., Desprez et al. 2014 at Macquarie Island). Uncertainty in the classification of breeding state can, however, be considered under several capture-recapture frameworks, including closed (Kendall et al. 2003, 2004) and open robust design (Ruiz-Gutierrez et al. 2016) models implemented in MARK, and multievent models (Pradel 2005) in implemented in E-SURGE. Here we show how the multievent-robust design model we fitted in the main text can be modified to include uncertainty in breeding state assignment during the breeding season.

*Modelling the state process*

The state process of the model do not change for a model with, compared to a model without, uncertainty. Females are assumed to occupy one of the following states each year:

1. breeder (B, pupped in the current year);
2. non-breeder (NB, pupped previously, but not in the current year);
3. dead (D, an absorbing state representing death and permanent emigration).

We present the transition and event matrices in the “GEPAT” convention of E-SURGE. Each row corresponds to a multinomial, a “-”represents a probability of 0, and a “*” means the complement to the probability on the same row. Probabilities are represented by letters (e.g., y, r), but transition probabilities need not be equal for similar notations.

Survival matrix:

Breeding transition matrix:

*Modelling the observation process*

When taking uncertainty of breeding state within the breeding season into account, there are 14 possible events which can be assigned to a female elephant seal and encoded in the encounter history matrix (Supporting Information 2: Table S1). Events are based on multiple capture periods in a year. Females can be encountered during three capture periods ($\kappa^{U},\kappa^{E},\kappa^{M}$), two of which ($\kappa^{U},\kappa^{E}$) corresponds to the robust design structure of the breeding season surveys.

The observation process occurring outside of the breeding season is defined first. The event matrix for $\kappa^{M}$ is

|  |  | B | B | NB | NB |
| --- | --- | --- | --- | --- | --- |
|  | NS | $M_{B}$ | $\bar{M_{B}}$ | $M_{NB}$ | $\bar{M_{NB}}$ |
| B | - | p | * | - | - |
| NB | - | - | - | p | * |
| DEAD | * | - | - | - | - |

This matrix gives the state-specific probability of capture during the moult and winter. Horizontal lines (bars) above column index labels denote ‘not seen’. Column 1 ($\mathrm{NS}$) represents dead individuals (never seen); column 2 ($M_{B}$) represents breeders detected in$\kappa^{M}$; column 3 ($\bar{M_{B}})$ represents breeders not seen in$\kappa^{M}$; column 4 ($M_{NB}$) represents non-breeders detected in$\kappa^{M}$; column 5 ($\bar{M_{NB}})$ represents non-breeders not seen in$\kappa^{M}.$

**Supporting Information S2: Table S1.** The 14 possible events assigned to a female elephant seal and encoded in the encounter history matrix based on multiple capture periods in a year. Events in bold (with event index followed by a ‘*’) indicate that a female was detected during the breeding season, but that her reproductive state was uncertain (i.e., her pup was not seen). Note that the new events (event codes 8 – 13) can arise for non-breeders as well as breeders, though with different probabilities. State assignment takes place in the fourth event matrix.

| Event code | Description | Pup status | Index |
| --- | --- | --- | --- |
| 0 | Not seen during any capture period | Pup seen | $\mathrm{NS}$ |
| 1 | Seen in all three capture periods ($\kappa^{U},\kappa^{E},\kappa^{M}$) | Pup seen | $\mathrm{UEM}$ |
| 2 | Seen in $\kappa^{U}$(breeding season ‘uneven’ sampling weeks) and $\kappa^{M}$ | Pup seen | $\mathrm{UM}$ |
| 3 | Seen in $\kappa^{E}$(breeding season ‘even’ sampling weeks) and $\kappa^{M}$ | Pup seen | $\mathrm{EM}$ |
| 4 | Only seen in $\kappa^{M}$(outside of the breeding season) | Pup seen | $M$ |
| 5 | Seen in $\kappa^{U}$ and $\kappa^{E}$ | Pup seen | $\mathrm{UE}$ |
| 6 | Only seen in $\kappa^{U}$ | Pup seen | $U$ |
| 7 | Only seen in $\kappa^{E}$ | Pup seen | $E$ |
| **8** | **Seen in all three capture periods (**$\boldsymbol{\kappa}^{\boldsymbol{U}}\boldsymbol{,}\boldsymbol{\kappa}^{\boldsymbol{E}}\boldsymbol{,}\boldsymbol{\kappa}^{\boldsymbol{M}}$**)** | **No pup** | $\mathbf{UEM}$***** |
| **9** | **Seen in** $\boldsymbol{\kappa}^{\boldsymbol{U}}$**(breeding season ‘uneven’ sampling weeks) and** $\boldsymbol{\kappa}^{\boldsymbol{M}}$ | **No pup** | $\mathbf{UM}$***** |
| **10** | **Seen in** $\boldsymbol{\kappa}^{\boldsymbol{E}}$**(breeding season ‘even’ sampling weeks) and** $\boldsymbol{\kappa}^{\boldsymbol{M}}$ | **No pup** | $\mathbf{EM}$***** |
| **11** | **Seen in** $\boldsymbol{\kappa}^{\boldsymbol{U}}$ **and** $\boldsymbol{\kappa}^{\boldsymbol{E}}$ | **No pup** | $\mathbf{UE}$***** |
| **12** | **Only seen in** $\boldsymbol{\kappa}^{\boldsymbol{U}}$ | **No pup** | $\mathbf{U}$***** |
| **13** | **Only seen in** $\boldsymbol{\kappa}^{\boldsymbol{E}}$ | **No pup** | $\mathbf{E}$***** |

Next, we define the observation process during$\kappa^{U}$, i.e., the ‘uneven’ survey weeks of the breeding season.

|  |  | B | B | B | B | NB | NB | NB | NB | NS |
| --- | --- | --- | --- | --- | --- | --- | --- | --- | --- | --- |
|  |  | $UM$ | $\bar{U}M$ | $U$ | $\bar{U}$ | $UM$ | $\bar{U}M$ | $U$ | $\bar{U}$ | NS |
| NS | NS | - | - | - | - | - | - | - | - | * |
| B | $M_{B}$ | p | * | - | - | - | - | - | - | - |
| B | $\bar{M_{B}}$ | - | - | p | * | - | - | - | - | - |
| NB | $M_{NB}$ | - | - | - | - | p | * | - | - | - |
| NB | $\bar{M_{NB}}$ | - | - | - | - | - | - | p | * | - |

In contrast to the model fitted in the main text, we assume here that non-breeders are potentially available for encounter in the breeding season. Rows 2 and 3 give the probability $\boldsymbol{p}^{\boldsymbol{u}}$ that a breeder was captured during at least one uneven survey week; its compliment ($\boldsymbol{1-p}^{\boldsymbol{u}}\boldsymbol{)}$is the probability of not being encountered during any of the $j$ weekly surveys of$\kappa^{U}$. Rows 4 and 5 refer to non-breeders.

The observation process during the ‘uneven’ survey weeks of the breeding season if followed by the observation process taking place in the ‘even’ survey weeks.

|  |  | B | B | B | B | B | B | B | NB | NB | NB | NB | NB | NB | NB |  |
| --- | --- | --- | --- | --- | --- | --- | --- | --- | --- | --- | --- | --- | --- | --- | --- | --- |
|  |  | $\mathrm{MUE}$ | $\mathrm{MU}$ | $\mathrm{ME}$ | $M$ | $\mathrm{UE}$ | $U$ | $E$ | $\mathrm{MUE}$ | $\mathrm{MU}$ | $\mathrm{ME}$ | $M$ | $\mathrm{UE}$ | $U$ | $E$ | NS |
| B | $UM$ | p | * | - | - | - | - | - | - | - | - | - | - | - | - | - |
| B | $\bar{U}M$ | - | - | p | * | - | - | - | - | - | - | - | - | - | - | - |
| B | $U$ | - | - | - | - | p | * | - | - | - | - | - | - | - | - | - |
| B | $\bar{U}$ | - | - | - | - | - | - | p | - | - | - | - | - | - | - | * |
| NB | $UM$ | - | - | - | - | - | - | - | p | * | - | - | - | - | - | - |
| NB | $\bar{U}M$ | - | - | - | - | - | - | - | - | - | p | * | - | - | - | - |
| NB | $U$ | - | - | - | - | - | - | - | - | - | - | - | p | * | - | - |
| NB | $\bar{U}$ | - | - | - | - | - | - | - | - | - | - | - | - | - | p | * |
| NS | NS | - | - | - | - | - | - | - | - | - | - | - | - | - | - | * |

Finally, in the state assignment matrix, the columns represent the events encountered in the field (Table A1.1). Breeders can be detected with or without pups in close proximity; non-breeders can only be encountered without a pup in close proximity.

|  |  | 0 | 1 | 2 | 3 | 4 | 5 | 6 | 7 | **8** | **9** | **10** | **11** | **12** | **13** |
| --- | --- | --- | --- | --- | --- | --- | --- | --- | --- | --- | --- | --- | --- | --- | --- |
|  |  | 0 | $\mathrm{MUE}$ | $\mathrm{MU}$ | $\mathrm{ME}$ | $M$ | $\mathrm{UE}$ | $U$ | $E$ | $\mathbf{MUE}$***** | $\mathbf{MU}$***** | $\mathbf{ME}$***** | $\mathbf{UE}$***** | $\mathbf{U}$***** | $\mathbf{E}$***** |
| B | $\mathrm{MUE}$ | - | * | - | - | - | - | - | - | p | - | - | - | - | - |
| B | $\mathrm{MU}$ | - | - | * | - | - | - | - | - | - | p | - | - | - | - |
| B | $\mathrm{ME}$ | - | - | - | * | - | - | - | - | - | - | p | - | - | - |
| B | $M$ | - | - | - | - | * | - | - | - | - | - | - | - | - | - |
| B | $\mathrm{UE}$ | - | - | - | - | - | * | - | - | - | - | - | p | - | - |
| B | $U$ | - | - | - | - | - | - | * | - | - | - | - | - | p | - |
| B | $E$ | - | - | - | - | - | - | - | * | - | - | - | - | - | p |
| NB | $\mathrm{MUE}$ | - | - | - | - | - | - | - | - | * | - | - | - | - | - |
| NB | $\mathrm{MU}$ | - | - | - | - | - | - | - | - | - | * | - | - | - | - |
| NB | $\mathrm{ME}$ | - | - | - | - | - | - | - | - | - | - | * | - | - | - |
| NB | $M$ | - | - | - | - | * | - | - | - | - | - | - | - | - | - |
| NB | $\mathrm{UE}$ | - | - | - | - | - | - | - | - | - | - | - | * | - | - |
| NB | $U$ | - | - | - | - | - | - | - | - | - | - | - | - | * | - |
| NB | $E$ | - | - | - | - | - | - | - | - | - | - | - | - | - | * |
|  | NS | * | - | - | - | - | - | - | - | - | - | - | - | - | - |

Literature cited

De Bruyn, P.J.N., Tosh, C.A., Bester, M.N., Cameron, E.Z., McIntyre, T. and Wilkinson, I.S. 2011. Sex at sea: alternative mating system in an extremely polygynous mammal. Animal Behaviour 82: 445–451.

Desprez, M., Harcourt, R., Hindell, M.A., Cubaynes, S., Gimenez, O. and McMahon, C.R. 2014. Age-specific cost of first reproduction in female southern elephant seals. Biology Letters 10: 20140264.

Kendall, W.L., Hines, J.E. and Nichols, J.D. 2003. Adjusting multistate capture-recapture models for misclassification bias: manatee breeding proportions. Ecology 84:1058-1066.

Kendall, W.L., Langtimm, C.A., Beck, C.A. and Runge, M.C. 2004. Capture-recapture analysis for estimating manatee reproductive rates. Marine Mammal Science 20: 424-437.

Pistorius, P.A., Bester, M.N., Kirkman, S.P. and Taylor, F.E. 2001b. Pup mortality in southern elephant seals at Marion Island. Polar Biology 24: 828–831.

Pradel, R. 2005. Multievent: an extension of multistate capture recapture models to uncertain states. Biometrics 61: 442-447.

Ruiz-Gutierrez, V., Kendall, W.L., Saracco, J.F. and White, G.C. 2016. Overwintering strategies of migratory birds: a novel approach for estimating seasonal movement patterns of residents and transients. Journal of Applied Ecology 53: 1035-1045.

**Supporting Information S3 - *Goodness-of-fit testing of southern elephant seal capture-recapture data at Marion Island***

Information-theoretic approaches to model selection (Burnham and Anderson 2002) assume that the set of models considered includes a general model that adequately fits the data. Lack-of-fit or overdispersion tends to inflate the deviance, erroneously favouring selection of overparametrized models (Pradel et al. 2005). Goodness of fit (GOF) testing is a generic problem with multiple approximate solutions, and no formal GOF tests exist for multievent- or open robust design models (Pradel et al. 2005).

We assumed that the estimate of overdispersion (ĉ) obtained from the Jolly-MoVe (JMV) multistate model (Pradel et al. 2003) applied to the models we fitted. We partitioned individual capture histories into distinct breeder and non-breeder components. For the purpose of goodness-of-fit testing we assumed that breeders were encountered in the breeding season and non-breeders were individuals only encountered outside of the breeding season, mainly in the moult. GOF tests were conducted using program U-CARE 2.2.2 (Choquet et al. 2009).

Capture histories displayed some departures of the model assumptions. “Newly marked” individuals in our sample (i.e., individuals entering the studied population at their first observed breeding attempt) were less likely to survive (and be re-encountered) than previously marked individuals (transience *sensu lato*, Test 3G.SR; Pradel et al. 2005). Secondly, individuals seen at occasion $t$were more likely to be re-encountered at $t+1$than individuals who were not seen at occasion $t$ (trap-happiness *sensu lato*, Test M.ITEC) (Supporting Information S3: Table S1). To account for the modest capture heterogeneity we adjusted parameter variances and model selection by using a variance inflation factor ($ĉ=\chi^{2}/df; ĉ$ $=$ 1.33) (Supporting Information S3: Table S1).

**Supporting Information S3: Table S1.** Testing the homogeneity assumptions of the Jolly-MoVe (JMV) multistate model for adult southern elephant seals at Marion Island, using component tests implemented in U-CARE.

| Test | $\chi^{2}$ | df^1^ | $ĉ$ |
| --- | --- | --- | --- |
| Test WBWA | 39.76 | 42 |  |
| Test 3G.SR | **49.13** | **26** |  |
| Test 3G.Sm | 107.47 | 131 |  |
| Test M.ITEC | **97.60** | **23** |  |
| Test M.LTEC | 13.64 | 10 |  |
| JMV Model | **307.60** | **232** | 1.33 |

^1^Degrees of freedom. Significant $\chi^{2}$ statistics (p < 0.05) are in boldface.

Literature cited

Burnham, K.P. and Anderson, D.R. 2002. Model selection and multimodel inference. A practical information-theoretic approach. 2nd edition. Springer Science and Business Media, Inc., New York, NY.

Choquet, R., Lebreton, J-D., Gimenez, O., Reboulet, A-M., Pradel, R. 2009b. U-CARE: Utilities for performing goodness of fit tests and manipulating capture-recapture data. Ecography 32: 1071-1074.

Pradel, R., Wintrebert, C.M.A. and Gimenez, O. 2003. A proposal for a goodness-of-fit test to the Arnason- Schwarz multistate capture-recapture model. Biometrics 59: 43-53.

Pradel, R., Gimenez, O. and Lebreton, J.D. 2005. Principles and interest of GOF tests for multistate capture-recapture models. Animal Biodiversity and Conservation 28: 189-204.

**Supporting Information S4 – *Multievent-robust design model structure with pre-breeders***

Observations of pre-breeders can easily be incorporated in the multievent robust-design framework.

*Modelling the state process*

Females are assumed to occupy one of the following states each year: pre-breeder (PB, has not previously pupped); breeder (B, pupped in the current year); non-breeder (NB, pupped previously, but not in the current year); and dead (D). The transition probabilities between states correspond to apparent annual survival ($\varphi$) and breeding probability ($\psi$). The state process can be decomposed as the product of a diagonal survival matrix by a conditional breeding probability matrix, with departure states in rows and arrival states in columns:

$$\left( \begin{matrix} \boldsymbol{\varphi}^{\boldsymbol{PB}} & \boldsymbol{0} & \boldsymbol{0} & \boldsymbol{1-}\boldsymbol{\varphi}^{\boldsymbol{PB}} \\ \boldsymbol{0} & \boldsymbol{\varphi}^{\boldsymbol{B}} & \boldsymbol{0} & \boldsymbol{1-}\boldsymbol{\varphi}^{\boldsymbol{B}} \\ \boldsymbol{0} & \boldsymbol{0} & \boldsymbol{\varphi}^{\boldsymbol{NB}} & \boldsymbol{1-}\boldsymbol{\varphi}^{\boldsymbol{NB}} \\ \boldsymbol{0} & \boldsymbol{0} & \boldsymbol{0} & \boldsymbol{1} \end{matrix} \right)\boldsymbol{\times}\left( \begin{matrix} \boldsymbol{1-}\boldsymbol{\psi}^{\boldsymbol{PB-B}} & \boldsymbol{\psi}^{\boldsymbol{PB-B}} & \boldsymbol{0} & \boldsymbol{0} \\ \boldsymbol{0} & \boldsymbol{\psi}^{\boldsymbol{B-B}} & \boldsymbol{1-}\boldsymbol{\psi}^{\boldsymbol{B-B}} & \boldsymbol{0} \\ \boldsymbol{0} & \boldsymbol{\psi}^{\boldsymbol{NB-B}} & \boldsymbol{1-}\boldsymbol{\psi}^{\boldsymbol{NB-B}} & \boldsymbol{0} \\ \boldsymbol{0} & \boldsymbol{0} & \boldsymbol{0} & \boldsymbol{1} \end{matrix} \right)$$

*Modelling the observation process*

We define the observation process occurring outside of the breeding season first. The event matrix for $\kappa^{M}$ is

| $\bar{M_{PB\&NB}}$ | $M_{B}$ | $\bar{M_{B}}$ | $M_{PB\&NB}$ |
| --- | --- | --- | --- |

$$\boldsymbol{B}^{\boldsymbol{M}}\boldsymbol{=}\begin{matrix} \mathrm{PB} \\ B \\ \mathrm{NB} \\ D \end{matrix}\left( \begin{matrix} \boldsymbol{1-}\boldsymbol{m}^{\boldsymbol{PB}} & \boldsymbol{0} & \boldsymbol{0} & \boldsymbol{m}^{\boldsymbol{PB}} \\ \boldsymbol{0} & \boldsymbol{m}^{\boldsymbol{B}} & \boldsymbol{1-}\boldsymbol{m}^{\boldsymbol{B}} & \boldsymbol{0} \\ \boldsymbol{1-}\boldsymbol{m}^{\boldsymbol{NB}} & \boldsymbol{0} & \boldsymbol{0} & \boldsymbol{m}^{\boldsymbol{NB}} \\ \boldsymbol{1} & \boldsymbol{0} & \boldsymbol{0} & \boldsymbol{0} \end{matrix} \right)$$

where $\boldsymbol{B}^{\boldsymbol{M}}$ is row-stochastic (i.e., probabilities in a row sum to one) with states in rows and events in columns. Matrix $\boldsymbol{B}^{\boldsymbol{M}}$ gives the state-specific probability of capture during the moult and winter. Horizontal lines (bars) above column index labels denote ‘not seen’. Column 1 ($\bar{M_{PB\&NB}}$) represents pre-breeders and non-breeders not seen in$\kappa^{M}$; column 2 ($M_{B}$) represents breeders detected in$\kappa^{M}$; column 3 ($\bar{M_{B}})$ represents breeders not seen in$\kappa^{M}$; and column 4 ($M_{PB\&NB}$) represents pre-breeders and non-breeders detected in$\kappa^{M}$. A pre-breeder therefore has a probability $\boldsymbol{m}^{\boldsymbol{PB}}$ to be seen during either the moult or the winter, and the complement probability ($\boldsymbol{1-m}^{\boldsymbol{PB}}$) to escape detection.

Because we assume in this model that neither pre-breeders nor non-breeders can be encountered in the breeding season, the two event matrices representing the robust-design capture periods of the breeding season do not change conditional on whether pre-breeders are excluded (main text) of included (this Supporting Information). The final two event matrices are thus the same as in the main text (barring row and column index labels).

Observation process during$\kappa^{U}$, i.e., the ‘uneven’ survey weeks of the breeding season:

| $UM$ | $\bar{U}M$ | $U$ | $\bar{U}$ | $\bar{M_{PB\&NB}}$ | $M_{PB\&NB}$ |
| --- | --- | --- | --- | --- | --- |

$$\boldsymbol{B}^{\boldsymbol{U}}= \begin{matrix} \bar{M_{PB\&NB}} \\ M_{B} \\ \bar{M_{B}} \\ M_{PB\&NB} \end{matrix}\left( \begin{matrix} \boldsymbol{0} & \boldsymbol{0} & \boldsymbol{0} & \boldsymbol{0} & \boldsymbol{1} & \boldsymbol{0} \\ \boldsymbol{p}^{\boldsymbol{u}} & \boldsymbol{1-p}^{\boldsymbol{u}} & \boldsymbol{0} & \boldsymbol{0} & \boldsymbol{0} & \boldsymbol{0} \\ \boldsymbol{0} & \boldsymbol{0} & \boldsymbol{p}^{\boldsymbol{u}} & \boldsymbol{1-}\boldsymbol{p}^{\boldsymbol{u}} & \boldsymbol{0} & \boldsymbol{0} \\ \boldsymbol{0} & \boldsymbol{0} & \boldsymbol{0} & \boldsymbol{0} & \boldsymbol{0} & \boldsymbol{1} \end{matrix} \right)$$

Observation process during the ‘even’ survey weeks of the breeding season:

| $\kappa$ | $\mathrm{NS}$ | $\mathrm{MUE}$ | $\mathrm{MU}$ | $\mathrm{ME}$ | $M$ | $\mathrm{UE}$ | $U$ | $E$ |
| --- | --- | --- | --- | --- | --- | --- | --- | --- |
| Event code | 0 | 1 | 2 | 3 | 4 | 5 | 6 | 7 |

$$\boldsymbol{B}^{\boldsymbol{E}}=\begin{matrix} UM \\ \bar{U}M \\ U \\ \bar{U} \\ \bar{M_{PB\&NB}} \\ M_{PB\&NB} \end{matrix}\left( \begin{matrix} \boldsymbol{0} & \boldsymbol{b}^{\boldsymbol{e}} & \boldsymbol{1-}\boldsymbol{b}^{\boldsymbol{e}} & \boldsymbol{0} & \boldsymbol{0} & \boldsymbol{0} & \boldsymbol{0} & \boldsymbol{0} \\ \boldsymbol{0} & \boldsymbol{0} & \boldsymbol{0} & \boldsymbol{b}^{\boldsymbol{e}} & \boldsymbol{1-}\boldsymbol{b}^{\boldsymbol{e}} & \boldsymbol{0} & \boldsymbol{0} & \boldsymbol{0} \\ \boldsymbol{0} & \boldsymbol{0} & \boldsymbol{0} & \boldsymbol{0} & \boldsymbol{0} & \boldsymbol{b}^{\boldsymbol{e}} & \boldsymbol{1-}\boldsymbol{b}^{\boldsymbol{e}} & \boldsymbol{0} \\ \boldsymbol{1-}\boldsymbol{b}^{\boldsymbol{e}} & \boldsymbol{0} & \boldsymbol{0} & \boldsymbol{0} & \boldsymbol{0} & \boldsymbol{0} & \boldsymbol{0} & \boldsymbol{b}^{\boldsymbol{e}} \\ \boldsymbol{1} & \boldsymbol{0} & \boldsymbol{0} & \boldsymbol{0} & \boldsymbol{0} & \boldsymbol{0} & \boldsymbol{0} & \boldsymbol{0} \\ \boldsymbol{0} & \boldsymbol{0} & \boldsymbol{0} & \boldsymbol{0} & \boldsymbol{1} & \boldsymbol{0} & \boldsymbol{0} & \boldsymbol{0} \end{matrix} \right)$$

**Supporting Information S5 – *Multievent model with state uncertainty and no robust design during the breeding season***

This multievent model has the exact same state process structure as described in the accompanying paper.

$$\left( \begin{matrix} \boldsymbol{\varphi}^{\boldsymbol{B}} & \boldsymbol{0} & \boldsymbol{1-}\boldsymbol{\varphi}^{\boldsymbol{B}} \\ \boldsymbol{0} & \boldsymbol{\varphi}^{\boldsymbol{NB}} & \boldsymbol{1-}\boldsymbol{\varphi}^{\boldsymbol{NB}} \\ \boldsymbol{0} & \boldsymbol{0} & \boldsymbol{1} \end{matrix} \right)\boldsymbol{\times}\left( \begin{matrix} \boldsymbol{\psi}^{\boldsymbol{B-B}} & \boldsymbol{1-}\boldsymbol{\psi}^{\boldsymbol{B-B}} & \boldsymbol{0} \\ \boldsymbol{\psi}^{\boldsymbol{NB-B}} & \boldsymbol{1-}\boldsymbol{\psi}^{\boldsymbol{NB-B}} & \boldsymbol{0} \\ \boldsymbol{0} & \boldsymbol{0} & \boldsymbol{1} \end{matrix} \right)$$

Thus, from an initial departure state ($\boldsymbol{\pi}_{\boldsymbol{t}}$) in the breeder state, individuals could be categorized with one of three distinct states:

(1) female was alive and breeding (line 1)

(2) female was alive and a non-breeder (line 2)

(3) female was dead (line 3).

The events for the multievent model were: not seen (0); seen breeding (1); and seen with an unknown breeding state (2). The breeding status of all other individuals observed only outside of the breeding season were considered to be unknown, and the model considered the probability that either the individual was non-breeder, or the individual was present and breeding, but escaped detection.

In the multievent model with no robust design structure in the breeding season, the encounter process was separated into two successive processes of detection $\boldsymbol{E}_{\boldsymbol{t}}$and breeding state assignment$\boldsymbol{A}_{\boldsymbol{t}}$, and given as the product of two matrices:

| $Not seen$ | $B$ | $NB$ | $0$ | 1 | $2$ |
| --- | --- | --- | --- | --- | --- |

$\boldsymbol{E}_{\boldsymbol{t}}\left( \begin{matrix} \boldsymbol{1-}\boldsymbol{p}_{\boldsymbol{B}} & \boldsymbol{p}_{\boldsymbol{B}} & \boldsymbol{0} \\ \boldsymbol{1-}\boldsymbol{p}_{\boldsymbol{NB}} & \boldsymbol{0} & \boldsymbol{p}_{\boldsymbol{NB}} \\ \boldsymbol{1} & \boldsymbol{0} & \boldsymbol{0} \end{matrix} \right)$ $\boldsymbol{\times}\boldsymbol{A}_{\boldsymbol{t}}\left( \begin{matrix} \boldsymbol{1} & \boldsymbol{0} & \boldsymbol{0} \\ \boldsymbol{0} & \boldsymbol{\delta}_{\boldsymbol{B}} & \boldsymbol{1-}\boldsymbol{\delta}_{\boldsymbol{B}} \\ \boldsymbol{0} & \boldsymbol{0} & \boldsymbol{1} \end{matrix} \right)$


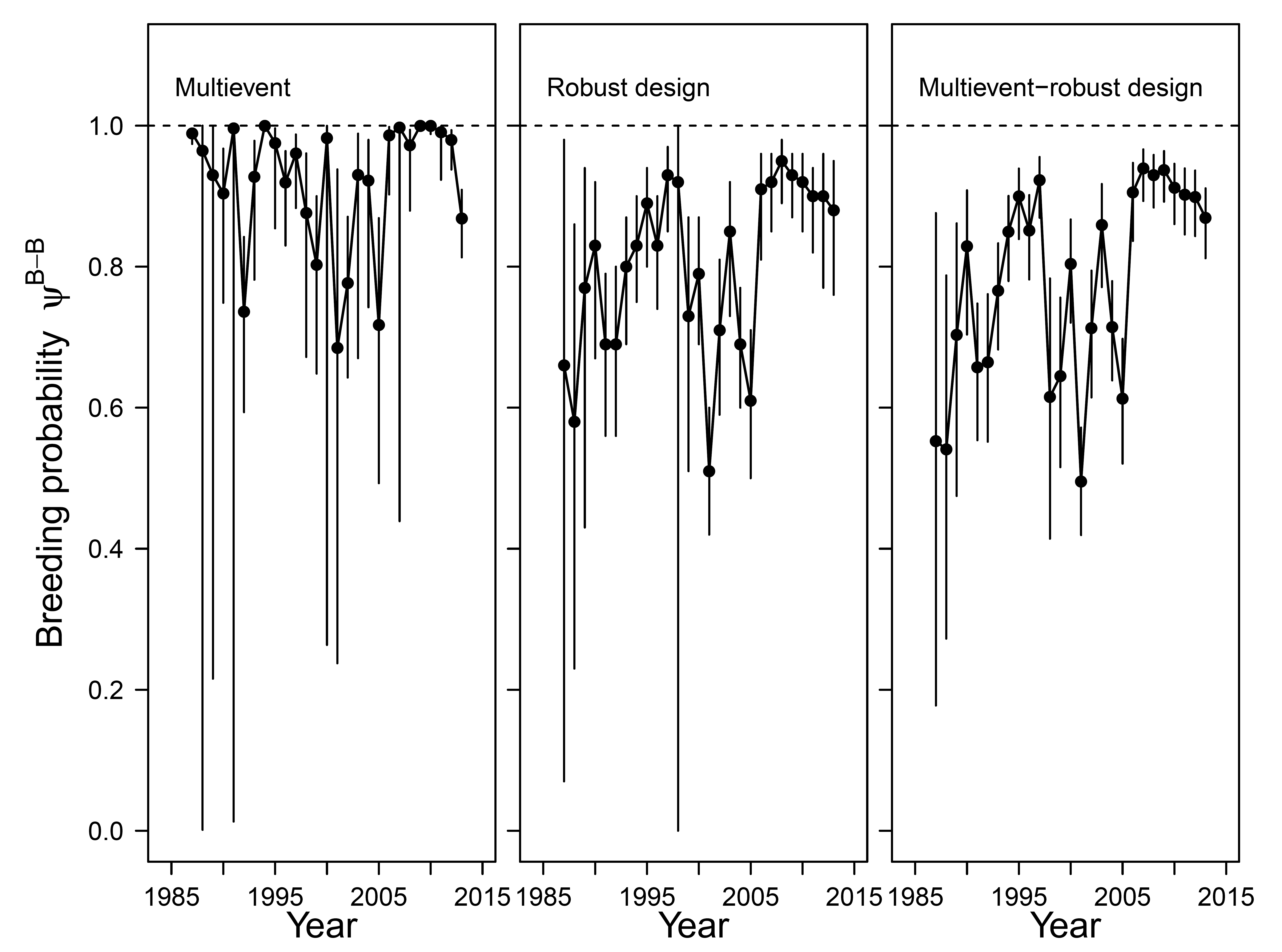


**Supporting Information S5: Fig S1.** Estimated probability (mean and 95% CI) of annual state-transition, from breeder to breeder ($\psi_{t}^{B-B}$) for female southern elephant seals at Marion Island (1986 – 2013) using three different analytic approaches. “Multievent” refers to a multievent model with state uncertainty but no robust design during the breeding season; “Robust design” refers to estimates obtained from a multistate open robust design model; and “Multievent-robust design” refers to the model described in the accompanying paper.


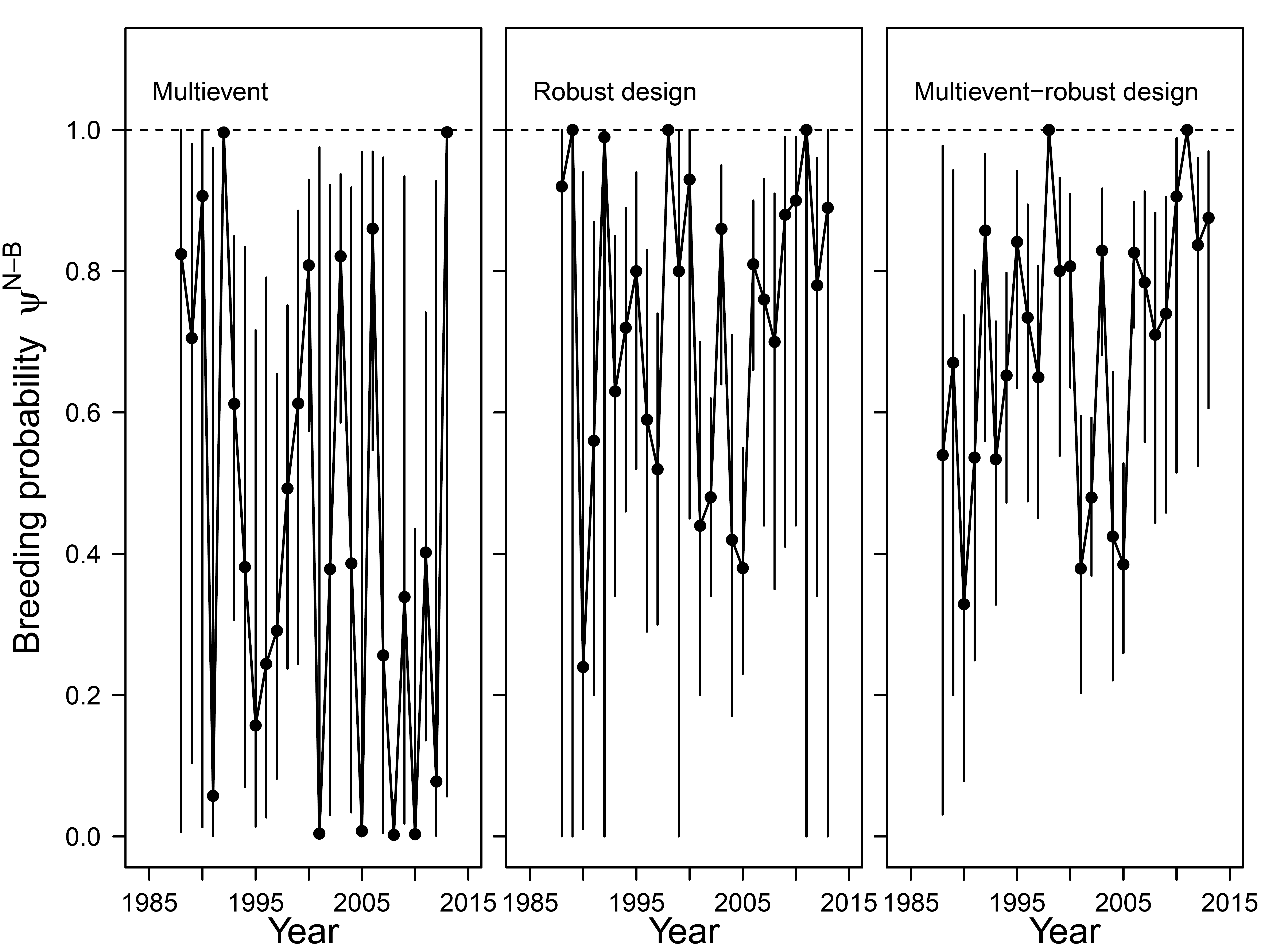


**Supporting Information S5: Fig S2.** Estimated probability (mean and 95% CI) of annual state-transition, from non-breeder to breeder ($\psi_{t}^{N-B}$) for female southern elephant seals at Marion Island (1986 – 2013) using three different analytic approaches. “Multievent” refers to a multievent model with state uncertainty but no robust design during the breeding season; “Robust design” refers to estimates obtained from a multistate open robust design model; and “Multievent-robust design” refers to the model described in the accompanying paper.

**Supporting Information S6 – *Multistate open robust design***

In this section we compare the demographic parameters obtained from the multievent-robust design model described in the accompanying paper to that derived from a multistate open robust design (MSORD) model (Kendall and Bjorkland 2001).

Multistate open robust design (MSORD) models permit the staggered arrival and departure of individual females through a breeding season by fitting the open-population model of Schwarz and Arnason (1996) at secondary occasions (Schwarz and Stobo 1997, Kendall and Bjorkland 2001). We built a MSORD model with $t = 28$ primary sampling periods (annual breeding seasons) with $j = 8$ secondary occasions (weekly island-wide surveys) per primary sampling period. Females were assumed to occupy one of two reproductive states each year: breeder (B) or non-breeder (NB). Surveys conducted outside of the breeding season did not contribute data to the MSORD analysis and therefore the non-breeder state was unobservable, i.e., non-breeders were temporary emigrants and always unavailable for capture.

Within primary periods we modelled $\beta_{j}^{B}$ as the probability that a breeder captured at secondary occasion $j$ is a new arrival to the breeding colony during that primary period; $\delta_{j,a}^{B}$ as the probability that a breeder at secondary occasion$j$, that arrived at previous secondary occasion$a$, is still at the breeding colony at$j + 1$; and $p_{t,j}^{B}$ as the probability that a breeder alive in year $t$ and present at the breeding colony at secondary occasion $j$ is detected. In good agreement with the breeding biology of elephant seals, the model allowed individuals to arrive and depart once during a primary period (Kendall and Bjorkland 2001). Entry probabilities $(\beta_{j}^{B})$ were allowed to be different between secondary occasions and we used a multinomial logit link function to ensure that the probability of entry across all eight surveys in a breeding season added to unity. The probability that a breeder will remain in the study area from one sampling period to the next $(\delta_{j,a}^{B})$ was modelled as a function of the number of sampling periods since it first arrived that season. Given strong inter-annual consistency in breeding phenology and the number of days it takes females to raise a pup, $\beta_{j}^{B}$and $\delta_{j,a}^{B}$were modelled as constant across all years. Capture probability was initially modelled as being fully time-dependent$(p_{t.t}^{B})$i.e., varying among years and among surveys within years. However, this resulted in a large number of parameters, many being estimated at the boundary (a capture probability of 0 or 1). We therefore simplified this parameter by assuming that capture probability varied only among years (i.e., constant across secondary occasions within a primary period $(p_{t}^{B}))$. We assumed that non-breeders were never observed and, therefore, set the probabilities$\beta_{j}^{N}$ $\delta_{j,a}^{N}$, and $p_{t,j}^{N}$to 0.

We fitted a similar set of models (Supporting Information S6: Table S1) compared to the multievent-robust design analyses described in the accompanying paper. In this case, however, because non-breeders were unobservable, we had to assume that breeders and non-breeders had the same apparent survival probability $(\varphi_{t}^{B}= \varphi_{t}^{NB})$ (Kendall and Bjorkland 2001). Program MARK 9.0 (White and Burnham 1999) was used to derive estimates of the parameters that maximize the likelihood of the observed data.

**Results**

Given that the MSORD could not include models with state-dependent survival, nearly all the MSORD model support ($w_{i}$ = 0.93) was apportioned to the model with the state-process $\varphi_{.} \psi_{t}^{B-B\neq NB-B}$(Supporting Information S6: Table S1). The survival probability of adult females estimated by the MSORD was identical (mean and variance, up to two decimal places) to that of the multievent robust design model, suggesting robust estimation. Breeder to breeder transition probability was also comparable between the two approaches (Supporting Information S6: Figure S1). In contrast, breeding probabilities of non-breeders displayed clear among-model variation (Supporting Information S6: Figure S1). The MSORD estimates of non-breeder breeding probabilities had lower precision in all years (Supporting Information S6: Fig. S2), and additionally included several estimates on the boundary. The relatively low proportion of non-breeders in the population gives rise to the generally poor precision of this parameter. In the MSORD model, the unobservable non-breeding state resulted in a further reduction in precision of non-breeder breeding probabilities.

**Supporting Information S6: Table S1.** Comparison of multistate open robust design (MSORD) models specifying different survival$(\varphi)$ and breeding $\left( \psi\right)$ probabilities of southern elephant seals at Marion Island (1986 - 2013). The number of parameters (np), model deviance, ΔQAIC (the difference in QAIC between the model with the lowest QAIC value and the relevant model) and the relative support by the data of a model, in relation to the other models (QAIC weight, $w_{i}$), is given. The model with support in the data is in boldface. Superscripts indicate state variation (𝐵 = breeder, 𝑁𝐵 = non-breeder), subscripts identify the absence (. ) or presence of time variation (𝑡).

| Model | np | Deviance | Δ QAIC | $w_{i}$ |
| --- | --- | --- | --- | --- |
| $\varphi_{t} \psi_{t}^{B-B\neq NB-B}$ | 114 | 37837.43 | 21.14 | 0.00 |
| $\boldsymbol{\varphi}_{\boldsymbol{.}} \boldsymbol{\psi}_{\boldsymbol{t}}^{\boldsymbol{B-B\neq NB-B}}$ | **93** | **37858.87** | **0.00** | **0.93** |
| $\varphi_{.} \psi^{B-B\neq NB-B}$ | 44 | 38070.47 | 112.70 | 0.00 |
| $\varphi_{.} \psi_{t}$ | 70 | 37910.54 | 5.17 | 0.07 |
| $\varphi_{.} \psi_{.}$ | 43 | 38102.55 | 142.77 | 0.00 |


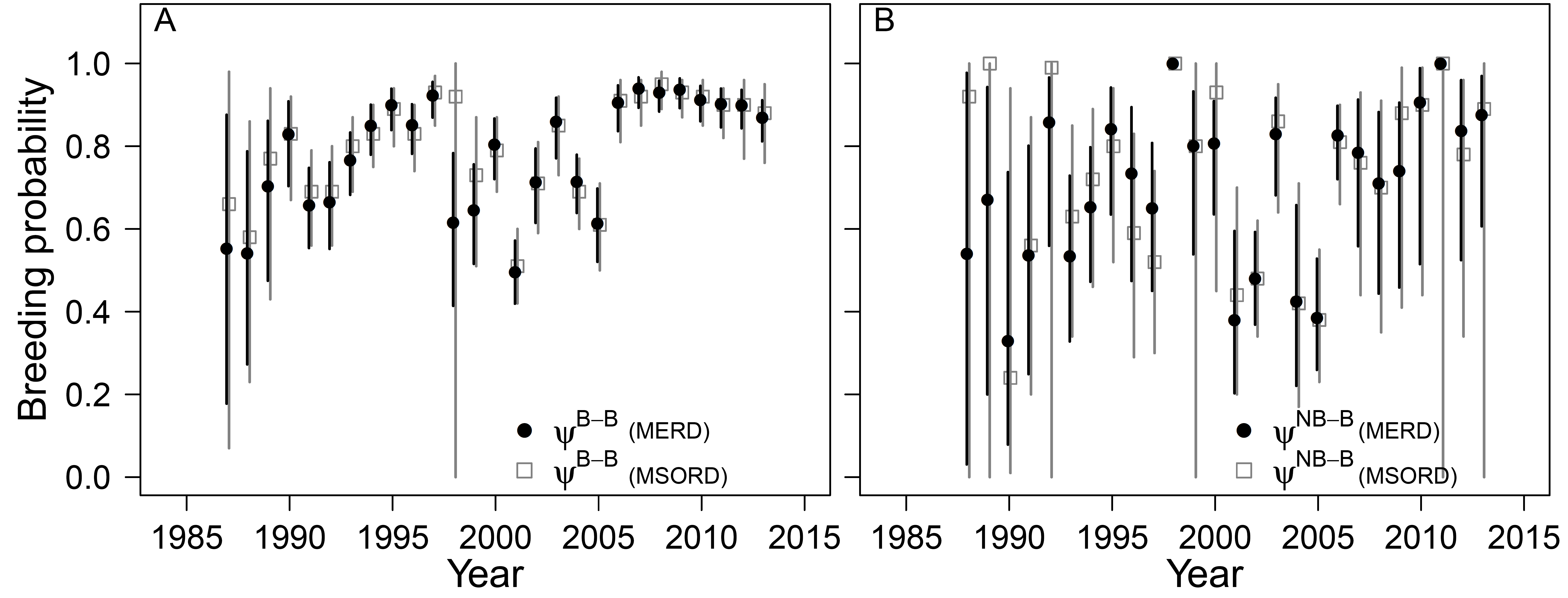


**Supporting Information S6: Fig. S1.** Annual breeding probability (mean and 95% confidence interval) of female elephant seals at Marion Island (1986 – 2013) estimated using multievent robust design (MERD, black circles) and multistate open robust design (MSORD, grey squares) models. (A) State-transition from breeder to breeder ($\psi_{t}^{B-B}$) (B) State-transition from non-breeder to breeder ($\psi_{t}^{NB-B}$).


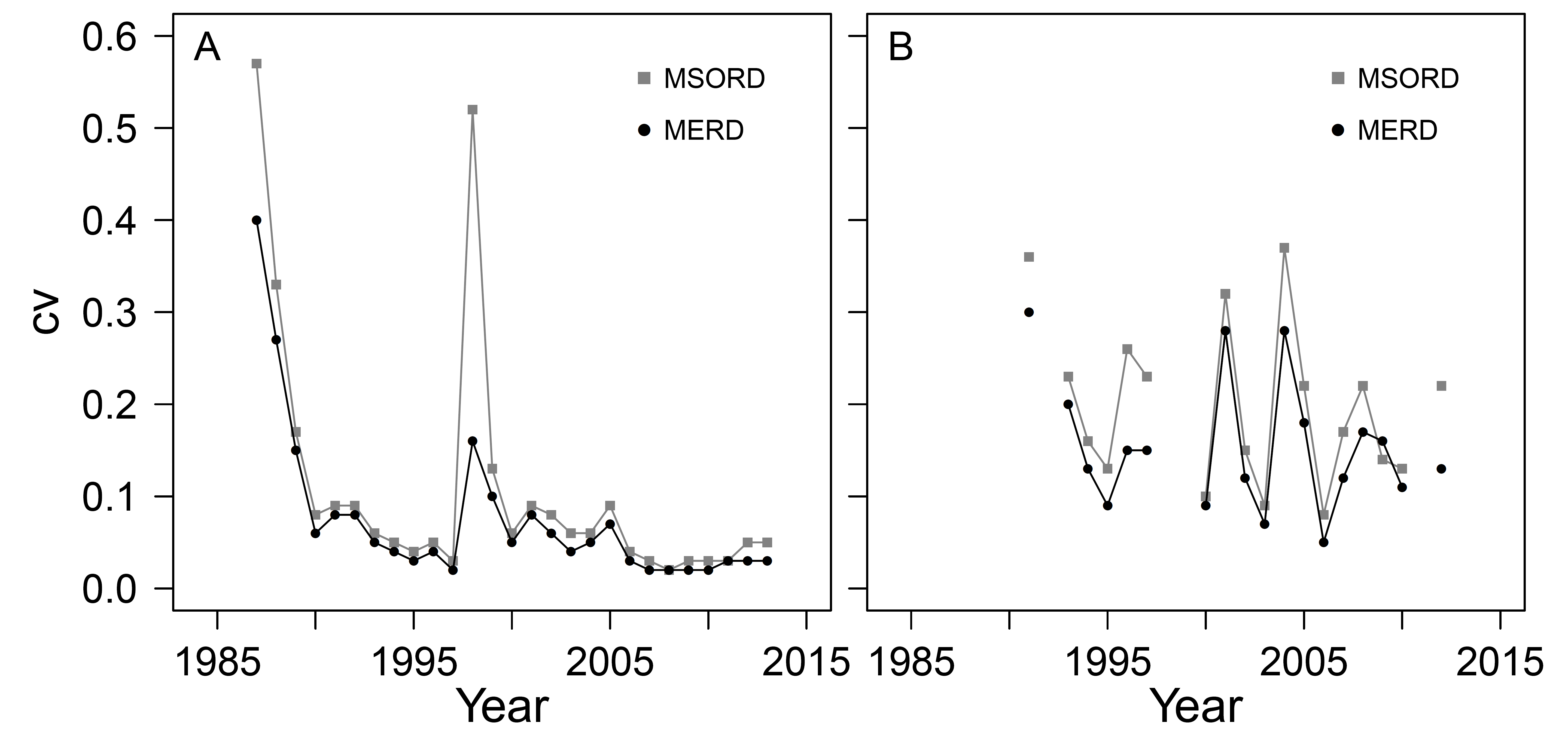


**Supporting Information S6: Fig. S2.** Comparing the coefficient of variation (cv) in estimated breeding probability among the multistate open robust design model (MSORD) and multievent-robust design model (MERD). Panel A. Transition from breeder to breeder. Panel B. Transition from non-breeder to breeder. Estimates for parameters on the boundary are not represented.

References

Kendall, W. L. and R. Bjorkland. 2001. Using open robust design models to estimate temporary emigration from capture–recapture data. Biometrics 57:1113-1122.

Schwarz, C. J. and A. N. Arnason. 1996. A general methodology for the analysis of capture recapture experiments in open populations. Biometrics 52: 860-873.

Schwarz, C. J. and W. T. Stobo. 1997. Estimating temporary migration using the robust design. Biometrics 53: 178-194.

White, G. C. and K. P. Burnham. 1999. Program MARK: survival estimation from populations of marked animals. Bird Study 46: S120-S139.

**Supporting Information S7 – *Derived parameters of the multistate open robust design model (MSORD)***

Multistate open robust design (MSORD) models allow several parameters to be derived: (1) the estimated number of marked individual females breeding in year$t$ $(\hat{N}_{t}^{B})$; (2) the average number of secondary sampling periods a breeder spent on land (residence time,$\hat{R}^{B}$); and (3) the effective capture probability ($p^{*}$), the probability of observing an individual at least once during a primary period.

For the Marion Island elephant seal MSORD analysis, the effective capture probability ${(p}^{*})$ was high during most years, poor detection in 1998 being a clear anomaly. But, detection of breeders was only ‘perfect’ $(p^{*} \to1)$ in less than half of all breeding seasons (Supporting Information S7: Fig. S1). Detection within each survey $j$ was clearly highest for the period 2006 to 2013, when field identification of all individuals present at the island during the breeding season can reasonably be assumed.


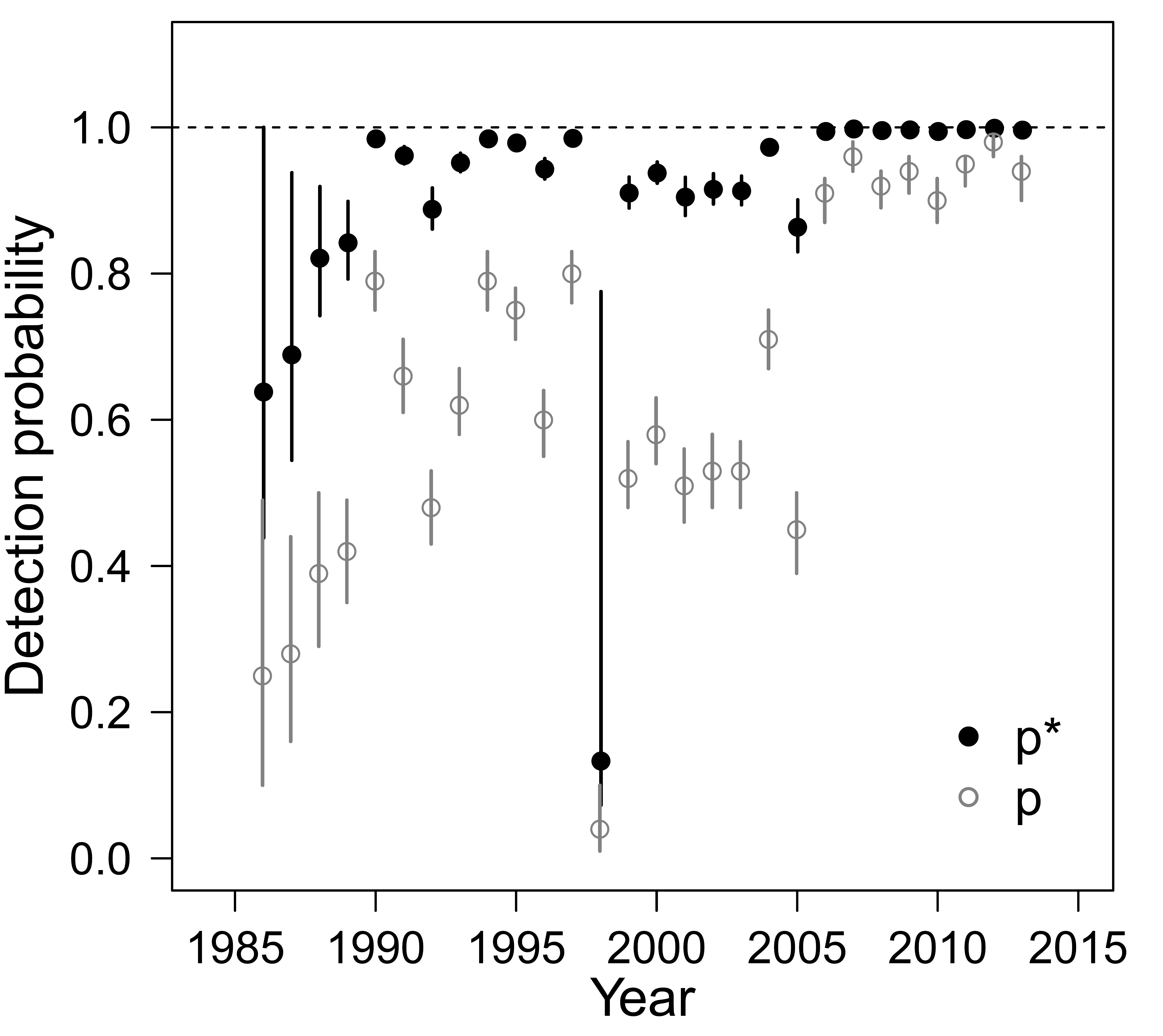


**Supporting Information S7: Fig. S1.** Estimated annual probability (mean and 95% CI) of detection of tagged female southern elephant seals during the breeding season at Marion Island (1986 – 2013). Open grey symbols ($p_{t}^{B}$): mean detection probability in each of eight secondary surveys conducted at weekly intervals. Solid black symbols ($p^{*}$): effective capture probability in the entire breeding season.

Given the generally high capture probability, model estimated abundance ($\hat{N}_{t}^{B})$ was usually close to the total number of individuals observed breeding (Supporting Information S7: Fig. S2). The expected residence time, as a function of time since arrival at the breeding colony (Supporting Information S7: Table S1), was $\hat{R}^{B}=$3.99 (3.95 - 4.03) weeks. The residence time estimated by the MSORD model agrees exactly with the known haulout duration of breeding female southern elephant seals: “28 days after arriving on the rookery, and on her last day of estrus, the female weans her pup by returning to sea” (Le Boeuf and Laws 1994).

**Supporting Information S7: Table S1.** Estimated parameters of movement to and from the study area for female southern elephant seals breeding at Marion Island (1986 - 2013). $\beta_{j}^{B}$: the probability of entering the study area during a particular secondary occasion. $\delta_{j,a}^{B}$: the probability that a breeder will remain in the study area from one weekly survey to the next.

| Sampling occasion | Probability of entry | | Probability of remaining | |
| --- | --- | --- | --- | --- |
|  | $\beta_{j}^{B}$ | 95% CI | $\delta_{j,a}^{B}$ | 95% CI |
| Survey 1 | 0.11 | 0.10 - 0.12 | 0.96 | 0.95 - 0.97 |
| Survey 2 | 0.21 | 0.19 - 0.22 | 0.99 | 0.97 - 1.00 |
| Survey 3 | 0.31 | 0.30 - 0.33 | 0.86 | 0.84 - 0.88 |
| Survey 4 | 0.24 | 0.22 - 0.25 | 0.26 | 0.24 - 0.28 |
| Survey 5 | 0.07 | 0.06 - 0.08 | 0.11 | 0.09 - 0.15 |
| Survey 6 | 0.05 | 0.04 - 0.06 | 0.32 | 0.18 - 0.50 |
| Survey 7 | 0.01 | 0.01 - 0.02 | 0.15 | 0.01 - 0.67 |
| Survey 8^1^ | 0.00 |  |  |  |

^1^ $\beta_{j=8}^{B}=(1-\sum\beta_{j=1,\ldots,7}^{B})$


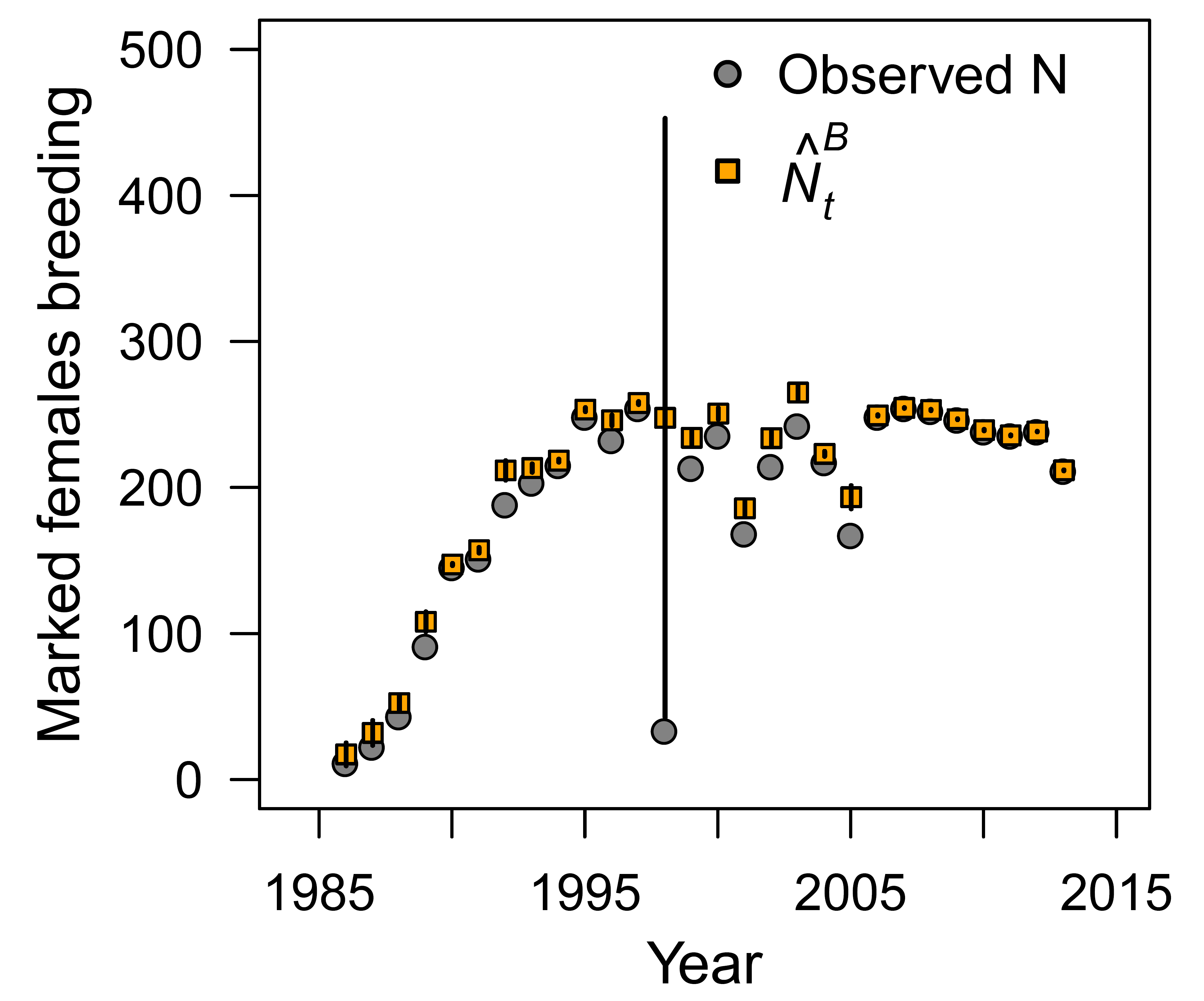


**Supporting Information S7: Fig. S2.** Estimated size of the marked breeding population of southern elephant seals at Marion Island, from 1986 to 2013. Grey circles: total number of marked individual females observed during each breeding season. Orange squares: estimated number of marked individual females breeding in year$t$, derived from the best fitting model. Bars are 95% confidence intervals.

Literature cited

Le Boeuf, B.J. and Laws, R.M. 1994. Elephant seals: an introduction to the genus. Pages 1-26 in Le Boeuf, B.J. and Laws, R.M. (Editors). Elephant seals: population ecology, behavior, and physiology. University of California Press, Berkeley, California, USA.
